# Applications of genome editing technology in the targeted therapy of human diseases: mechanisms, advances and prospects

**DOI:** 10.1038/s41392-019-0089-y

**Published:** 2020-01-03

**Authors:** Hongyi Li, Yang Yang, Weiqi Hong, Mengyuan Huang, Min Wu, Xia Zhao

**Affiliations:** 1 Department of Gynecology and Obstetrics, Development and Related Disease of Women and Children Key Laboratory of Sichuan Province, Key Laboratory of Birth Defects and Related Diseases of Women and Children, Ministry of Education, West China Second Hospital, Sichuan University, Chengdu, 610041 P. R. China; 2 Laboratory of Aging Research and Cancer Drug Target, State Key Laboratory of Biotherapy, National Clinical Research Center for Geriatrics, West China Hospital, Sichuan University, No. 17, Block 3, Southern Renmin Road, Chengdu, Sichuan 610041 P. R. China; 3 Department of Biomedical Sciences, School of Medicine and Health Sciences, University of North Dakota, Grand Forks, ND 58203 USA

**Keywords:** Genetic techniques, Gene therapy

## Abstract

Based on engineered or bacterial nucleases, the development of genome editing technologies has opened up the possibility of directly targeting and modifying genomic sequences in almost all eukaryotic cells. Genome editing has extended our ability to elucidate the contribution of genetics to disease by promoting the creation of more accurate cellular and animal models of pathological processes and has begun to show extraordinary potential in a variety of fields, ranging from basic research to applied biotechnology and biomedical research. Recent progress in developing programmable nucleases, such as zinc-finger nucleases (ZFNs), transcription activator-like effector nucleases (TALENs) and clustered regularly interspaced short palindromic repeat (CRISPR)–Cas-associated nucleases, has greatly expedited the progress of gene editing from concept to clinical practice. Here, we review recent advances of the three major genome editing technologies (ZFNs, TALENs, and CRISPR/Cas9) and discuss the applications of their derivative reagents as gene editing tools in various human diseases and potential future therapies, focusing on eukaryotic cells and animal models. Finally, we provide an overview of the clinical trials applying genome editing platforms for disease treatment and some of the challenges in the implementation of this technology.

## Introduction

**Fig. 1:**
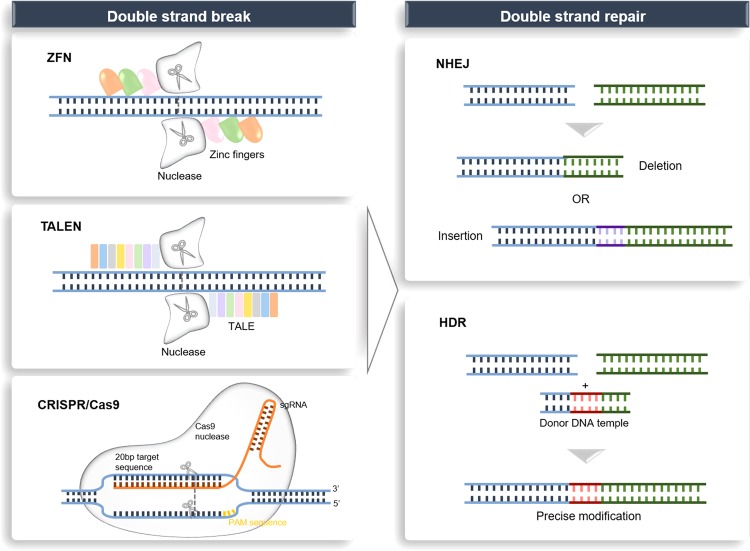
Genome editing platforms and mechanisms for DSB repair with endogenous DNA. Genome editing nucleases (ZFNs, TALENs and CRISPR/Cas9) induce DSBs at targeted sites. DSBs can be repaired by NHEJ or, in the presence of donor template, by HDR. Gene disruption by targeting the locus with NHEJ leads to the formation of indels. When two DSBs target both sides of a pathogenic amplification or insertion, a therapeutic deletion of the intervening sequences can be created, leading to NHEJ gene correction. In the presence of a donor-corrected HDR template, HDR gene correction or gene addition induces a DSB at the desired locus. DSB double-stranded break, ZFN zinc-finger nuclease, TALEN transcription activator-like effector nuclease, CRISPR/Cas9 clustered regularly interspaced short palindromic repeat associated 9 nuclease, NHEJ nonhomologous end-joining, HDR homology-directed repair.

Over the last few years, the exuberant development of genome editing has revolutionized research on the human genome, which has enabled investigators to better understand the contribution of a single-gene product to a disease in an organism. In the 1970s, the development of genetic engineering (manipulation of DNA or RNA) established a novel frontier in genome editing.^1^ Based on engineered or bacterial nucleases, genome editing technologies have been developed at a rapid pace over the past 10 years and have begun to show extraordinary utility in various fields, ranging from basic research to applied biotechnology and biomedical research.^2^ Genome editing can be achieved in vitro or in vivo by delivering the editing machinery in situ, which powerfully adds, ablates and “corrects” genes as well as performs other highly targeted genomic modifications.^3,4^ Targeted DNA alterations begin from the generation of nuclease-induced double-stranded breaks (DSBs), which leads to the stimulation of highly efficient recombination mechanisms of cellular DNA in mammalian cells.^5,6^ Nuclease-induced DNA DSBs can be repaired by one of the two major mechanisms that occur in almost all cell types and organisms: homology-directed repair (HDR) and nonhomologous end-joining (NHEJ),^7^ resulting in targeted integration or gene disruptions, respectively (Fig. 1).

Historically, homologous recombination (HR), in which undamaged homologous DNA fragments are used as templates, has been the approach to realize targeted gene addition, replacement, or inactivation; however, the utility of HR is heavily limited due to its inefficiency in mammalian cells and model organisms.^8^ After it was discovered that DSBs could raise the incidence of HDR by multiple orders of magnitude, targeted nucleases have been found as an alternative approach to increase the efficiency of HDR-mediated genetic alteration. Once a targeted DSB has been made, HDR may reconstruct the cleaved DNA using an exogenous DNA template analog to the break site sequence.

This mechanism may be used to introduce precise mutations by delivering an appropriately designed repair template into targeted cells directly,^9,10^ thereby, in a site-specific manner, resulting in mutation correction or new sequence insertion. Alternatively, NHEJ-mediated repair tends to result in errors because it leads to efficient formation of gene insertion or deletion (indels) in diverse lengths at the DSB site, which eventually causes gene inactivation.^11^ If indels occur in the coding sequence, there will be frameshift mutations, which will result in mRNA degradation or nonfunctional truncated protein production by nonsense-mediated decay.^12^ This approach and its applications are thought to be simpler than HR-based methods because (a) there is no need for a repair matrix and (b) the cell type has less impact on modification efficacy (contrary to HR, NHEJ may be active all through the cell cycle).^13^ Thus, similar to RNAi, NHEJ may be applied in immortalized cell lines to generate the inactivation of a single gene or multiple genes, but by creating loss-of-function mutations, it may lead to permanent gene inactivation.^9^

In the early development stage of genome editing, to induce the desired DSBs at each particular DNA target site, the engineering of distinct zinc-finger nucleases (ZFNs)^14^ or meganucleases^15^ has been the research focus. These nuclease systems required specialized competence to generate artificial proteins consisting of customizable sequence-specific DNA-binding domains, each connected to a nonspecific nuclease for target cleavage, providing researchers with unprecedented tools to perform genetic manipulation.^16^ Subsequently, a new class of a *Flavobacterium okeanokoites* (FokI) catalytic domain derived from bacterial proteins termed transcription activator-like effectors (TALEs) has shed light on new possibilities for precise genome editing.^17^ TALE-based programmable nucleases can cleave any DNA sequence of interest with relatively high frequency. However, the main challenges for transcription activator-like effector nucleases (TALEN) approaches are the design of a complex molecular cloning for each new DNA target and its low efficiency of genome screening in successfully targeted cells.^18^ Clustered regularly interspaced short palindromic repeat (CRISPR)-associated 9 (Cas9) nuclease is a recently discovered, robust gene editing platform derived from a bacterial adaptive immune defense system.^19^ This system can be efficiently programmed to modify the genome of eukaryotic cells via an RNA-guided DNA cleavage module and has emerged as a potential alternative to ZFNs and TALENs to induce targeted genetic modifications^20^ (Table 1). Since 2013, when it was first applied in mammalian cells as a tool to edit the genome,^21,22^ the versatile CRISPR/Cas9 technology has been rapidly expanding its use in modulating gene expression, ranging from genomic sequence correction or alteration to epigenetic and transcriptional modifications.

**Table 1:** Comparison of ZFN, TALEN and CRISPR/Cas9 platforms.

	ZFN	TALEN	CRISPR/Cas9
Recognition site	Zinc-finger protein	RVD tandem repeat region of TALE protein	Single-strand guide RNA
Modification pattern	Fok1 nuclease	Fok1 nuclease	Cas9 nuclease
Target sequence size	Typically 9–18 bp per ZFN monomer, 18–36 bp per ZFN pair	Typically 14–20 bp per TALEN monomer, 28–40 bp per TALEN pair	Typically 20 bp guide sequence + PAM sequence
Specificity	Tolerating a small number of positional mismatches	Tolerating a small number of positional mismatches	Tolerating positional/multiple consecutive mismatches
Targeting limitations	Difficult to target non-G-rich sites	5ʹ targeted base must be a T for each TALEN monomer	Targeted site must precede a PAM sequence
Difficulties of engineering	Requiring substantial protein engineering	Requiring complex molecular cloning methods	Using standard cloning procedures and oligo synthesis
Difficulties of delivering	Relatively easy as the small size of ZFN expression elements is suitable for a variety of viral vectors	Difficult due to the large size of functional components	Moderate as the commonly used SpCas9 is large and may cause packaging problems for viral vectors such as AAV, but smaller orthologs exist

*ZFN* Zinc-finger nuclease, *TALEN* Transcription activator-like effector nuclease, *CRISPR* Clustered regularly interspaced short palindromic repeat

The advent of programmable nucleases has greatly accelerated the proceedings of gene editing from concept to clinical practice and unprecedentedly enabled scientists with a powerful tool to maneuver literally any gene in a wide variety of cell types and species. Current preclinical research on genome editing primarily concentrates on viral infections, cardiovascular diseases (CVDs), metabolic disorders, primary defects of the immune system, hemophilia, muscular dystrophy, and development of T cell-based anticancer immunotherapies. Some of these methods have gone beyond preclinical research and are recently undergoing phase I/II clinical trials. Here, we review recent improvements of the three main genome editing platforms (ZFN, TALENs, and CRISPR/Cas9) and discuss applications of their derivative reagents as gene editing tools in various human diseases and in promising future therapies, focusing on eukaryotic cells and animal models. Finally, we outline the clinical trials applying genome editing platforms for disease treatment and some of the challenges in the implementation of this technology.

## Structure and mechanism of genome editing tools

### The structure of ZFNs and their interaction with DNA

ZFNs are assembled by fusing a non-sequence-specific cleavage domain to a site-specific DNA-binding domain that is loaded on the zinc finger.^23^ The zinc-finger protein with site-specific binding properties to DNA was discovered primarily in 1985 as part of transcription factor IIIa in *Xenopus* oocytes.^24^ The functional specificity of the designed zinc-finger domain comprises an array of Cys_2_His_2_ zinc fingers (ZFs), which are derived by highly conserved interactions of their zinc-finger domains with homologous DNA sequences. Generally, an individual Cys_2_His_2_ zinc finger consists of approximately 30 amino acids, which constitute two anti-parallel β sheets opposing an α-helix.^25^ Cys_2_-His_2_-ZF is an adaptable DNA recognition domain and is considered to be the most common type of DNA-binding motif in eukaryotic transcription factors.^26^ Each zinc-finger unit selectivity recognizes three base pairs (bp) of DNA and produces base-specific contacts through the interaction of its α-helix residues with the major groove of DNA.^27,28^ The FokI type II restriction endonuclease forms the domain that cleaves the DNA, which can be adopted as a dimer to directly target sequences within the genome for effective gene editing.^29^ Since the FokI nuclease needs to be dimerized to cleave DNA, two ZFN molecules are usually required to bind to the target site in an appropriate orientation,^30^ doubled in the number of specifically recognized base pairs. After DNA cleavage by ZFNs is achieved in eukaryotic cells, DSBs at a specific locus of the genome is initiated, creating the desired alterations in subsequent endogenous NHEJ or HDR repair systems.^23^

The target sequence recognition and specificity of ZFNs are determined by three major factors: (a) the amino acid sequence of each finger, (b) the number of fingers, and (c) the interaction of the nuclease domain. By virtue of the modular structure of ZFNs, both the DNA-binding and catalytic domains can be individually optimized, which enables scientists to develop novel modular assembly with sufficient affinity and specificity for genome engineering. In early studies, individual ZFNs containing 3–6 fingers were used to interact with a 9–18 nucleotide target, which enabled ZFN dimers to specify 18–36 bp of DNA at each cleavage site.^31^ Since the 18 bp sequence of DNA can render specificity within 68 billion bp of DNA, this approach facilitated the targeting of specific sequences in the human genome for the first time. A more recently developed strategy used architectural diversification to improve the targeting accuracy of ZFNs via “selection-based methods”^32^: this study developed a new linker option for spanning finger–finger and finger–FokI cleavage domain junctions, which produced a 64-fold total increase in the number of ZFN configurations available for targeting cleavage to any given base of DNA.

### TALENs: a protein-based DNA targeting system

TALENs are another type of engineered nuclease that exhibit better specificity and efficiency than ZFNs. Similar to ZFNs, TALENs comprise a nonspecific DNA cleavage domain fused to a customizable sequence-specific DNA-binding domain to generate DSBs. This DNA-binding domain consists of a highly conserved repeat sequence from transcription activator-like effector (TALE), which is a protein originally discovered in the phytopathogenic *Xanthomonas* bacteria that naturally alters the transcription of genes in host plant cells.^17,33^ The binding of TALE to DNA is mediated by a central region that contains an array of 33- to 35-amino-acid sequence motifs. The amino acid sequence of each repeat is structurally similar, except for two hypervariable amino acids (the repeat variable di-residues or RVDs) at positions 12 and 13.^34^ DNA-binding specificity is determined by RVDs, with ND specifically binding to C nucleotides, HN to A or G nucleotides, NH to G nucleotides, and NP to all nucleotides.^17^ There is a one-to-one correspondence between RVDs and contiguous nucleotides in the target site, constituting a strikingly simple TALE–DNA recognition cipher.^35^

Functional endonuclease FokI is factitiously fused to DNA-binding domains to create site-specific DSBs and thereby stimulate DNA recombination to achieve TALEN-induced targeted genomic modification. To cleave the two strands of the targeted DNA, the FokI cleavage domain must be dimerized. Hence, like zinc fingers, such a TALEN module is designed in pairs to bind opposing DNA target loci, with proper spacing (12–30 bp) between the two binding sites.^36^ However, compared to zinc-finger proteins, there is no need to redesign the linkage between repeats constituting long arrays of TALEs, which function to target individual genomic sites. Following pioneering works on zinc-finger proteins, multiple effector domains have become accessible to support the fusion of TALE repeats for different genomic modification purposes, including nucleases,^37^ transcriptional activators,^18^ and site-specific recombinases.^38^ Although their simpler cipher codes provide better simplicity in design than triplet-confined zinc-finger proteins, one of the primary technical hurdles for cloning repeat TALE arrays is the design of a large scale of identical repeat sequences. To address this limitation, a few strategies have been established to facilitate the fast assembly of custom TALE arrays, including “Golden Gate” molecular cloning,^39^ high-throughput solid phase assembly,^40,41^ and connection-independent cloning techniques.^42^

### CRISPR/Cas9: a versatile tool for genome editing

Early in 1987, clustered regularly interspersed short palindromic repeats (CRISPRs) were originally discovered in *E. coli* and later in many other bacteria species.^43^ The function of the short repeat sequences remained unclear for many years before several studies in 2005 characterized their similarities to phage DNA, and subsequent experiments revealed that these sequences took part in bacterial and archaea adaptive immune defense against offending foreign DNA by inducing RNA-guided DNA cleavage.^44–46^ Generally, the CRISPR‐Cas systems are divided into two classes based on the structural variation of the *Cas* genes and their organization style.^44^ Specifically, class 1 CRISPR–Cas systems consist of multiprotein effector complexes, whereas class 2 systems comprise only a single effector protein; altogether, six CRISPR-Cas types and at least 29 subtypes have been reported,^47,48^ and the list is rapidly expanding. The most frequently used subtype of CRISPR systems is the type II CRISPR/Cas9 system, which depends on a single Cas protein from *Streptococcus pyogenes* (SpCas9) targeting particular DNA sequences and is therefore an attractive gene editing tool.^49^ Mechanistically, the CRISPR/Cas9 system comprises two components, a single-stranded guide RNA (sgRNA) and a Cas9 endonuclease. The sgRNA often contains a unique 20 base-pair (bp) sequence designed to complement the target DNA site in a sequence-specific manner, and this must be followed by a short DNA sequence upstream essential for the compatibility with the Cas9 protein used, which is termed the “protospacer adjacent motif” (PAM) of an “NGG” or “NAG”.^50,51^ The sgRNA binds to the target sequence by Watson–Crick base pairing and Cas9 precisely cleaves the DNA to generate a DSB.^52^ Following the DSB, DNA-DSB repair mechanisms initiate genome repair. With the CRISPR/Cas9 system, through pathways of NHEJ or high-fidelity HDR, targeted genomic modifications, including the introduction of small insertions and deletions (indels), can be made.^53^

Known as the RNA‐guided system, CRISPR/Cas9 is more suitable for application compared to other gene editing technologies and has several important advantages.^20^ For example, endonuclease-based ZFN or TALEN tools demand reengineering of the enzyme to fit each target sequence, and they should be synthesized separately for each case; however, the nuclease protein Cas9 is identical in all cases and can be conveniently engineered to recognize new sites via changing the guide RNA sequences (sgRNA), which match target sites by Watson–Crick base pairing. Moreover, in contrast to CRISPR/Cas9, ZFNs and TALENs demand much more labor and are more expensive. An additional advantage of CRISPR/Cas9 is that it has the potential of simultaneous multiple loci editing, making this technology easier, more efficient, and more scalable compared to other genome editing technologies. CRISPR/Cas9 is now an indispensable tool in biological research.

Three common strategies have been developed for genome editing with the CRISPR/Cas9 platform: (1) the plasmid‐based CRISPR/Cas9 strategy, where a plasmid is used to encode Cas9 protein and sgRNA,^21,22^ assembles Cas9 gene as well as sgRNA into the same plasmid in vitro. this strategy is longer lasting in the expression of Cas9 and sgRNA, and it prevents multiple transfections.^54^ However, the encoded plasmid needs to be introduced inside the nucleus of target cells, which is a key challenge in this system; (2) direct intracellular delivery of Cas9 messenger RNA (mRNA) and sgRNA,^55^ the greatest drawback of which lies in the poor stability of mRNA, which results in transient expression of mRNA and a short duration of gene modification; (3) directly delivery of Cas9 protein and sgRNA^56^, which has several advantages, including rapid action, great stability, and limited antigenicity.

The editing of DNA means the irreversible permanent change of genome information, and this process is also facing inevitable security risks and ethical problems. In addition, some cell types, such as neurons, are difficult to modify DNA using CRISPR/Cas9-mediated editing, which limits the use of gene therapy for nervous system diseases. As a result, genome editing strategies that only edit and modify RNA have also been proposed by scientists.^57,58^ As an intermediate product of DNA transcription, RNA is responsible for guiding the production of downstream proteins. With the use of CRISPR technology, RNA mutation is modified briefly, which not only avoids the irreversible modification of the genome but also can repair protein function in almost all cells to treat a variety of diseases. Stem cell transplantation combined with the CRISPR/Cas9 system is another approach for the therapy of genetic mutations. It has been proven that patient-induced pluripotent stem cells (iPSCs) have the ability to differentiate into retinal precursors, and it is a useful cell source for cell replacement therapy without immune rejection problems.^59,60^ However, patient-derived iPSCs might still harbor the same pathogenic genes, which could influence the therapeutic efficacy of transplanted cells. Therefore, it is necessary to combine the CRISPR/Cas9 system to fix disease-causing mutations in patient-derived iPSCs before transplantation.^61^

## Genome editing for disease modeling and gene therapy

**Fig. 2:**
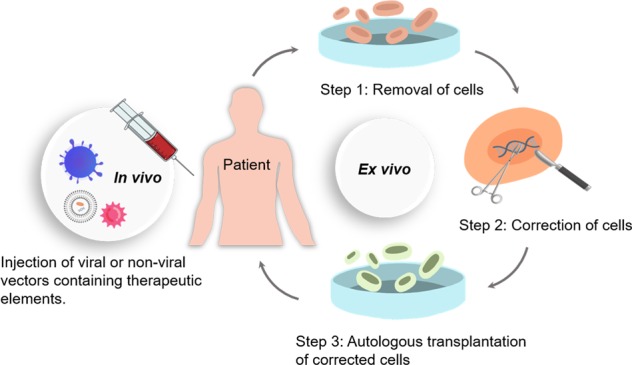
Ex vivo and in vivo genome editing for clinical therapy. Right: For in ex vivo editing therapy, cells are isolated from a patient to be treated, edited and then re-engrafted back to the patient. To achieve therapeutic success, the target cells must be able to survive in vitro and return to the target tissue after transplantation. Left: For in vivo editing therapy, engineered nucleases are delivered by viral or nonviral approaches and directly injected into the patient for systemic or targeted tissue (such as the eye, brain, or muscle) effect.

Targeted gene modification via chimeric genome editing tools (e.g., ZFNs, TALENs, and CRISPR/Cas9) is a powerful method to assess gene function and precisely manipulate cellular behavior and function. These genome editing tools have enabled investigators to use genetically engineered animals to understand the etiology behind various diseases and to clarify molecular mechanisms that can be exploited for better therapeutic strategies (Fig. 2).

### Cancer research

Oncogenes and mutant tumor suppressor genes provide outstanding opportunities for the use of genome modulating approaches.^62^ Genome editing technology has accomplished crucial targeted cleavage events in various fundamental studies, from its initial proofs of efficient gene editing in eukaryotes to its recent applications in the engineering of hematopoietic stem cells (HSCs) and tumor-targeted T cells; this technology has established novel concepts of gene modification and has extended to a border field of cancer research.

As an archetypal platform for programmable DNA cleavage, ZFN-mediated targeting has been successfully applied to modify many genes in human cells and a number of model organisms, thus opening the door to the development and application of genome editing technologies. ZFN-driven gene disruption was primarily demonstrated in 1994 when a three-finger protein was constructed to specifically block the expression of the BCR-ABL human oncogene that was transformed into a mouse cell line.^63^ After that, a study used a human lymphoblast cell line derived from chronic myeloid leukemia (CML) patients, and a custom-designed ZFN was applied to this cell line to deliver site-specific DSBs to the telomeric portion of the mixed lineage leukemia (MLL) gene breakpoint cluster region as well as to analyze chromosomal rearrangements associated with MLL leukemogenesis via DSB error repair.^64^ Successful targeted modulation was also achieved using designed ZFNs, which promoted the disruption of endogenous T cell receptor (TCR) β- and α-chain genes. Lymphocytes treated with ZFNs lacked the surface expression of CD3-TCR and expanded with an increase in interleukin-7 (IL-7) and IL-15.^65^ By targeting the promoter function of long terminal repeat (LTR) from human T cell leukemia virus type 1 (HTLV-1), a novel therapeutic ZFN specifically killed HTLV-1-infected cells in an in vivo model of adult T cell leukemia (ATL).^66^ In addition, it was reported that effective cleavage of the BCR-ABL fusion gene by highly specific ZFNs terminated the translation of the BCR-ABL protein and induced apoptosis in imatinib-resistant CML cells.^67^ Furthermore, cancer-relevant translocations in human Ewing sarcoma and anaplastic large cell lymphoma (ALCL) cells induced by ZFNs demonstrated that precise genomic rearrangements can be achieved in relevant cell types by custom nucleases.^68^ Furthermore, the use of HER2-positive cell-penetrating peptide (CPP) conjugated to mammalian mTOR-specific ZFN made the mTOR locus nonfunctional and inhibited relevant cancer signaling pathways, providing insight into the design of novel molecular targeted therapeutics for breast cancer (in particular) and other types of cancers.^69^ Moreover, as the tumor suppressor gene p53 plays a pivotal role in preventing cancer development, strategies of genome editing to restore wild-type p53 function have been investigated. A yeast-one-hybrid (Y1H) four-finger ZFN was designed to replace mutant p53 with wild-type p53 in several cancer cell lines (from glioblastoma, leukemia and breast cancer) via ZFN-induced HR.^70^ Although the HR events were not particularly effective in this case, modifications at p53 loci still provided a framework for further investigation. In addition to modifying viral genes associated with tumorigenesis, researchers have applied ZFNs to optimize T cell-mediated antitumor therapy. For example, by importing a chimeric TCR that comprises an extracellular IL-13 domain (zetakine) and a cytoplasmic CD3 domain into CD8 + T cells, glioblastoma-specific cytolytic T lymphocytes (CTLs) can be generated. To achieve this goal, Reik et al.^71^ knocked down the glucocorticoid receptor in the modified CTLs with ZFNs. Consequently, the cytolytic activity of “zetakine” transgenic CTLs against glioblastomas was preserved regardless of the presence of glucocorticoid treatment. This technology has recently been effective in knocking out glucose transport-related genes (MCT4 or BSG) in two glycolytic tumor models: colon adenocarcinoma and glioblastoma.^72^

A milestone of TALENs was achieved when they were primarily applied to efficiently disrupt the endogenous genes NTF3 and CCR5 in human leukemia cells via the introduction of NHEJ- or HDR-induced modification into a coding sequence, demonstrating that TALENs could be designed for selective endogenous gene cleavage.^73^ Interestingly, when TALENs and ZFNs were compared abreast at two human loci (CCR5 and IL2RG), TALENs showed a significant reduction in cytotoxicity. Moreover, the CCR5-specific TALEN was able to distinguish between the CCR5 target locus and a highly similar site in CCR2 when compared with ZFN technology.^37^ By adopting TALEN gene editing technology, precise disruptions have also been introduced into the T cell receptor α constant (TRAC) gene and the CD52 gene in allogeneic T cells by TALEN-induced HDR. The TALEN used in this study was engineered by a retroviral vector that expressed a chimeric antigen receptor (CAR) targeting CD19+ leukemic B cells, which helped to develop the “universal” CAR T cells (dKO-CART19).^74^ Alternatively, a site-specific TALEN was used to disrupt a single allele of the Fms-related tyrosine kinase 3 (FLT3) gene and generate isogenic leukemia cell clones. TALEN-mediated FLT3 haplo-insufficiency impaired cell proliferation and colony formation in vitro. These suppressive effects were maintained in vivo and improved the survival rate of NOD/SCID mice transplanted with mutant K562 clones.^75^ The use of engineered TALENs in prostate cancer cells functionally classifies androgen receptor (AR) target gene rearrangements as drivers of resistance.^76^ Using TALENs to precisely cut the relevant translocation breakpoints, Piganeau et al. induced cancer-relevant translocations in anaplastic large cell lymphoma (ALCL).^68^ Through an analogous strategy, the reversion of ALCL translocation was achieved in a patient cell line, restoring the integrity of the two involved chromosomes. Recent studies have also shown that TALEN gene editing technology used to knock out genes in cancer cells (including cells from prostate cancer,^76^ breast cancer,^77^ and hepatocellular carcinoma (HCC)^78^) is a powerful and broadly applicable platform to explore gene mutations at the molecular level.

Because of its multiple advantages in genome editing, the CRISPR/Cas9 system has attracted considerable attention, and scientists gradually consider it to be a powerful therapeutic tool for treating diseases associated with genome mutations. The ultimate goal of cancer therapy with CRISPR/Cas9 is to remove malignant mutations and replace them with normal DNA sequences.^79^ In a recent study, the leukemia model was generated by reviving several inactivated oncogenes through the lentiviral delivery of the Cas9-sgRNA system in primary hematopoietic stem and progenitor cells (HSPCs).^80^ In this study, the pooled lentiviruses targeted genes, including Tet2, Runx1, Dnmt3a, Nf1, Ezh2, and Smc3. The objective HSPCs were selected via a fluorescent marker; those HSPCs are engaged in the development of myeloid neoplasia. CRISPR/Cas9 technology has also been adopted to establish organoid tumor models.^81,82^ For instance, organoid colon cancer models were constructed in vitro with CRISPR technology by introducing mutations of tumor suppressor genes (APC, TP53, SMAD4, etc.) and gene modification of oncogenes (KRAS, PI3K, etc.).^83^ Moreover, guided by colonoscopy, through mucosal injection, Roper et al.^84^ established CRISPR engineered mouse tumor organoids by delivering viral vectors carrying CRISPR/Cas9 components to the distal colon of mice. Such an approach has already been applied in a study modeling tumor progression with an adenoma-carcinoma-metastasis sequence. In the future, the use of CRISPR/Cas9 technology to establish precise cancer models will significantly promote the research of functional cancer genomics and facilitate the advancement of cancer therapies.

### Cardiovascular disease

CVD is a serious hazard to human health and is the number one cause of death in many industrialized countries. Many different types of CVD are usually associated with a single genetic mutation or a combination of rare inherited heterozygous mutations.^85^ In practice, clinical treatments focus on the relief of disease symptoms without addressing potential genetic defects. Currently, the establishment of in vivo CVD models with gene editing technology and the in-depth analysis of CVD pathogenic genes as well as their molecular mechanisms have made it possible to test the ability of gene therapy to control specific gene expression and improve gene functions. With the help of genome editing technologies, various research models of cardiovascular conditions have been created.

Abrahimi et al.^86^ used CRISPR/Cas9 to efficiently ablate major histocompatibility complex class II (MHCII) with double gene knockout in normal human endothelial cells. These cells retain the ability to form vascular structures without activating allogeneic CD4+ T cells. It is promising to apply such technology in the field of allograft bioengineering, including the refinement of heart transplant. In addition, CRISPR/Cas9 technology can accurately remove β2M and CCR5 on CD34+ HSCs while retaining its ability to undergo multidifferentiation, which provides the possibility for the future treatment of ischemic heart conditions with HSCs.^86^ In another study, Carroll et al.^87^ established a cardiac-specific transgenic mouse model by injecting Cas9-containing plasmids into mouse zygotes; the expression of Cas9 was regulated by the Myh6 promoter. In this transgenic model, high levels of Cas9 were expressed exclusively in heart cardiomyocytes. The investigators then intraperitoneally injected sgRNA targeting Myh6 loaded in an adeno-associated virus (AAV) vector, subsequently inducing cardiac-specific gene modification at the Myh6 locus, finally leading to hypertrophic cardiomyopathy.

It has been demonstrated in the whole-exome sequencing of a nuclear family that three missense variants of a single nucleotide in the MKL2, MYH7, and NKX2-5 genes pass on to three offspring with cardiomyopathy with childhood onset.^88^ Gifford et al.^89^ adopted CRISPR/Cas9 to establish a mouse model that encodes orthologous variants and showed that the complex of heterozygosity of all three variants reproduced the phenotype of human disease. An analysis of mouse heart and human induced pluripotent stem-cell-derived cardiomyocytes provides histological and molecular evidence for the contribution of the NKX2-5 variant as a genetic modifier.

Porcine models resemble human conditions by physiology, anatomy, and genetics and are often considered ideal models for human cardiovascular structure research. Yang et al.^90^ applied ZFN technology with nuclear transfer in somatic cells to generate endogenous gene knockout pigs, which have a specific mutation in peroxisome proliferator-activated receptor gamma (PARP-γ). Marfan syndrome (MFS) is an autosomal dominant disease caused by a mutation of heterozygous fibrillin-1 (FBN1) and presents cardiovascular symptoms and skeletal abnormalities. By the same principle, Umeyama et al.^91^ accomplished the establishment of FBN1 mutant cloned pigs (+Glu433AsnfsX98), which exhibited phenotypes similar to those of humans with MFS, such as scoliosis, funnel chest, delayed epiphysis mineralization, and the destruction of elastic fiber structure in the medial aortic tissue.

Human induced pluripotent stem cells (iPSCs) and CRISPR/Cas9 technology can also be combined to generate a congenital heart disease model associated with GATA4 mutations in vitro to investigate the pathogenesis of this gene mutation.^92,93^ Using Barth syndrome (BTHS) iPSC-derived cardiomyocytes (iPSC-CMs) and genome editing, Wang and colleagues demonstrated that TAZ mutation is associated with myocardial metabolism and structural and functional abnormalities.^93^ These findings indicate the value of genetically edited animals as models for research on the pathogenesis of CVD and provide new insights into treatment strategies.

By genome editing techniques, potential therapeutic methods of repairing disease-causing mutations or of knocking out specific genes as CVD prevention approaches have also received widespread attention. For example, long QT syndrome (LQTS) is an autosomal dominant congenital heart disease. Hybrid mutations in multiple genes may lead to LQTS, some of which have relatively clear mutation sites with known molecular functions, such as hERG gene mutations in the pore-forming subunit alpha protein that encodes the potassium voltage-gated channel. The hERG gene mainly expresses and functions in cells of the myocardium and smooth muscle, and its mutation can cause fatal ventricular arrhythmia.^94^ Repairing hERG gene mutations in cardiomyocytes using CRISPR technology may be an effective strategy to treat such LQTS.

Previous studies have noted that nonsense mutation carriers of the proprotein convertase subtilisin/kexin type 9 (PCSK9) gene have significantly decreased levels of low-density lipoprotein cholesterol (LDL-C) in their blood compared with normal subjects (an allelic mutation corresponds to a 30 to 40% reduction).^95^ The blood level of triglyceride (TG) in subjects with nonsense mutations in the apolipoprotein C3 (APOC3) gene was significantly lower than that in unaffected people (an allelic mutation corresponds to a 40% decrease).^96^ The incidence of heart disease in both carriers was lower than that in unaffected subjects by more than 80%, suggesting that the inhibition of PCSK9 and APOC3 gene expression can be used as a potential treatment for cardiovascular disease. Since these two genes are mainly expressed in liver cells, one idea is to directly introduce nonsense mutations to APOC3 or PCSK9 genes in liver cells through genome editing technology, thus fundamentally inhibiting protein synthesis and achieving long-term stable therapeutic effects.^97,98^

PRKAG2 cardiac syndrome is an autosomal dominant disease induced by a mutation in the PRKAG2 gene encoding the AMP-activated protein kinase γ2 regulatory subunit. A recent study suggests that the selective destruction of pathogenic mutations through CRISPR/Cas9 technology in vivo is a competent strategy to treat PRKAG2 heart syndrome and other dominant hereditary heart conditions.^99^

### Metabolic diseases

Metabolic diseases refer to the pathological state in which the body’s protein, fat, carbohydrates, etc. are metabolically disordered. Metabolic diseases include a group of syndromes that are caused by both genetic factors and the environment.^100^ Gene editing technology can be applied in functional gene screening, gene therapy and the construction of metabolic disease models, such as obesity, diabetes, and hyperlipidemia. Leptin (Lep) is a hormone secreted by white fat cells that acts on the metabolic regulation center of the hypothalamus through the leptin receptor (LepR).^101^ It has diverse functions, including appetite suppression, energy intake reduction, and fat synthesis inhibition, and can regulate blood sugar concentration, neuroendocrine, etc. A number of animal models have been developed to illustrate the important role of Lep/LepR in glycolipid metabolism, and the most widely used are ob/ob mice against Lep and db/db mice against LepR.^102^ Chen and colleagues injected TALEN components into rat zygotes to specifically knockout LepR, thus obtaining three lines of rats with LepR mutations.^103^ Phenotypes in these strains manifested as obesity and other metabolic disorders; additionally, the authors established a LepR mutant obese rat model, exhibiting efficient germline transmission. Bao et al.^104^ successfully established LepR knockout mice using CRISPR/Cas9 technology. Homozygous LepR-deficient mice are characterized by obesity, hyperphagia, hyperglycemia, insulin resistance, and lipid metabolism disorders, together with some complications of diabetes. The same principle has been used to generate the cytochrome P450 (CYP) 2E1 knockout rat model with CRISPR/Cas9 technology to explore the role of the CYP2E1 gene in biochemical metabolism, toxicology, and diseases (e.g., diabetes and alcoholic cirrhosis).^105^ The FTO allele is associated with obesity, which inhibits the mitochondrial thermogenic effects in adipose precursor cells. FTO gene mutations inhibit the conversion of white fat to brown fat. The FTO gene-regulated thermogenic pathway involves ARID5B, rs1421085, IRX3, and IRX5 factors. rs1421085 can be edited using the CRISPR/Cas9 platform to repair the pattern structure of ARID5B, thereby suppressing the expression of IRX3 and IRX5 and achieving the effect of weight loss.^106^

As an important “experimental tool”, the animal model of diabetes can be used for pathological observation, preclinical experiments and drug screening. In a study based on CRISPR/Cas9 technology, pX330 (containing gRNA and Cas9 sequences together with the donor DNA plasmid) was injected into the oocyte to generate new Cre tool mice and achieve the genetic manipulation of pancreatic β cells.^107^ The Ins1 (insulin gene) promoter and stop codon sequences served as targets for recombinase Cre insertion. Progeny F1 mice were histologically labeled as Cre-loxP recombination, which was observed in all islets expressing insulin-positive cells and negatively expressed in other tissues. There was no significant difference in glucose tolerance between these genetically edited mice and wild-type mice. Applying CRISPR/Cas9 technology in human iPSCs to target diabetes-related genes has become a promising approach to explore the molecular mechanisms of diabetes. For example, human iPSCs are isolated from single-gene diabetic MODY patients, and possible mutations in genes such as HNF4A, GCK, PDX-1, and INS are edited by CRISPR; the edited iPSCs then differentiate into pancreatic progenitor cells and are later transplanted into patients.^108^ In addition, gene editing tools can also structurally modify proteins that promote chromatin structural variation, such as methylase, demethylase, acetylase or deacetylase, to treat diabetes epigenetically.^109^

Gene editing technology is also critically involved in the study of lipid metabolism.^110^ cAMP responsive element binding protein 3-like 3 (CREB3L3), a transcription factor expressed in the liver and small intestine, controls the energy metabolic equilibrium in fasting response. Nakagawa et al.^111^ used the one-step CRISPR/Cas9 system to establish the CREB3L3-floxed murine model for the first time and subsequently obtained mice that were knocked out of the CREB3L3 gene in the small intestine and liver, respectively. The evidence above provides a new understanding of the role of CREB3L3 in plasma triglyceride metabolism and its contribution to liver and intestinal cholesterol metabolism. Familial hypercholesterolemia is an autosomal single-gene dominant disease correlated with a defect in the low-density lipoprotein receptor (LDLR) gene, which causes a disorder of the body’s lipid metabolism. In 2012, Carlson et al.^112^ used TALEN technology to target LDLR in porcine fetal fibroblasts and obtained miniature swine containing mono- and biallelic mutations in LDLR, thus generating models of familial hypercholesterolemia, which came with critical biomedical significance in simulating lipid metabolic syndrome. Recent genome-wide association studies have identified tribble homolog 1 (TRIB1) to be associated with lipoprotein metabolism in human hepatocytes. Hepatic-specific overexpression of Trib1 reduced plasma TG and cholesterol levels by reducing the production of VLDL; in contrast, Trib1-knockout mice showed elevated plasma TG and cholesterol levels due to the increased production of VLDL.^113^ To further explore its regulation of lipid metabolism, Nagiec et al.^114^ induced the destruction of the chromosome at the TRIB1 locus by delivering the CRISPR/Cas9 system into mouse liver via a nonpathogenic AAV, which increased the transcription of PCKS9 and the secretion of PCKS9 protein; these responses ultimately reduced the level of liver LDL receptors and increased the level of LDL-C in the blood.

### Neurodegenerative diseases

Neurodegenerative diseases (NDs), at least including Huntington’s disease (HD), Alzheimer’s disease (AD), and Parkinson’s disease (PD), are a group of conditions that have attracted the most concern because there have been no specific diagnostic approaches or established treatments for them.^115,116^ There are a few potential pathogenic mechanisms behind NDs, including the accumulation of proteins with abnormal structures,^117^ impaired ubiquitin-proteasome and/or autophagic lysosomal pathways,^118^ oxidative stress^119^ and circuit alternations^120^, etc. These mechanisms indicate that NDs are induced by complicated interactions of multiple genetic factors; either alone or in combination, the interactions lead to clinical features. The emergence of gene editing platforms provides a convenient approach to study gene functions related to NDs.^121^

In HD, in vitro investigations demonstrated that via ZFNs, chromosomal expression of the mutant huntingtin (HTT) gene was significantly reduced at both the protein and mRNA levels; in vivo studies revealed that via striatal AAV delivery into the HD R6/2 mice, ZFNs extensively suppressed cerebral expression of the HTT gene and ameliorated HD-related symptoms.^122^ Additionally, the HTT exon 1 in human iPSCs derived from fibroblasts of HD patients (HD-iPSCs) can be corrected by TALENs.^123,124^ To better understand the pathogenesis of HD, Yan et al.^125^ adopted CRISPR/Cas9 to establish a genome-edited porcine model of HD in 2018, which internally expressed full-length mutant HTT. As a promising breakthrough in the field of NDs, the development of HTT gene knock-in pigs would be of great significance for pathogenesis research and therapy exploration in Huntington disease.

Mutations in the gene encoding amyloid precursor protein (APP) cause familial AD with nearly complete penetrance.^126^ Mouse fibroblast cells overexpress APP by receiving electroporated ZFNs designed with a DNA fragment containing the promoter and the protein coding regions of APP. These transgenic cells can be used to elucidate aspects of the molecular mechanisms of AD pathogenesis, particularly those involved in the mutant amyloidogenic pathway affecting the APP coding sequence.^127^ The A673V variant near the APP β-secretase cleavage site contributes to AD pathology by increasing Aβ and enhancing its aggregation as well as toxicity;^128^ by contrast, the A673T variant, which is adjacent to the aspartyl protease β-site in APP, provides protection against AD progression.^129^ When A673V and A673T were induced in normal iPSCs by TALEN technology, these cells differentiated and formed cortical neurons, presenting with different levels of AD-associated biomarkers.^130^ In addition, through a gene editing platform based on single-stranded oligonucleotide DNA nucleotides and CRISPR/CAS-blocking mutations, Paquet et al.^131^ generated human iPSCs with dominant AD-causing mutations in APP and presenilin 1 (PSEN1), both heterozygous and homozygous, leading to early disease onset; thereby, they yielded cortical neurons, which showed genotype-dependent phenotypes associated with AD. Apolipoprotein E4 (APOE4) is a genetic risk factor for late-onset AD, while ApoE2, which differs from APOE4 by only two bases (two C bases in APOE4, corresponding to two U bases in APOE2), is not a risk factor for AD. Zhang and his team introduced APOE4 RNA related to disease risk into cells and successfully changed the APOE4 to APOE2 sequence through the RESCUE (RNA Editing for Specific C to U Exchange) editing system by changing two C bases in APOE4, which is equivalent to converting the disease risk of the AD high-risk population carrying the APOE4 gene to zero.^132^

Alpha-synuclein (SNCA) and leucine-rich repeat kinase 2 (LRRK2) are associated with autosomal dominant PD, whereas another group of genes are associated with autosomal-recessive PD, including parkin, phosphatase and tensin homolog–induced kinase 1 (PINK1), DJ-1, and ATPase type 13A2 (ATP13A2).^133^ The missense mutation of SNCA and LRRK2 genes can be corrected by ZFNs in vitro. After correction, the mtDNA damage disappeared in differentiated neural progenitor and neural cells derived from iPSCs.^134,135^ Additionally, Soldner et al.^136^ combined genome-wide epigenetic information with CRISPR/Cas9 genome editing to generate a genetically precisely controlled experimental system in human iPSCs. This system has identified PD-associated risk variants in noncoding distal enhancer elements that regulate SNCA expression; it has also confirmed that the transcriptional disorder of SNCA is related to sequence-dependent binding of the brain-specific transcription factors EMX2 and NKX6-1. These results suggest that gene editing techniques can generate specific ND animal models for further exploration into human diseases, and they are potentially capable of offering a robust therapeutic approach against multiple human genetic defects that have been considered incurable.

### Viral diseases

Gene editing platforms have emerged recently as antiviral therapeutics for treating infectious diseases, either by altering the host genes required by the virus or by targeting the viral genes necessary for replication.^137^ To date, genome editing-based HIV therapy has involved modifying infection-related genes to produce HIV-resistant CD4+ T cells and subsequently reinfusing the edited cells into patients. In 2008, the anti-HIV efficacy of the ZFN system was first presented in preclinical studies by adopting primary human CD4+ T cells.^138^ Approximately 50% of the CCR5 alleles were disrupted with ZFN, which was delivered by the chimeric Ad5/F35 adenoviral vector. HIV-infected mice transfused with ZFN-modified CD4+ T cells also better preserved their original CD4+ T cells and had lower viral loads than nontransfused mice. In 2009, a patient was functionally cured of HIV infection by transplanting allogeneic stem cells from a donor with a homozygous CCR5 d32 allele,^139^ suggesting that it is feasible to obtain resistance to HIV by mimicking natural homozygous CCR5 d32 mutations with genome editing technologies. In addition, engineering CD34+ HSPCs instead of CD4+ T cells with the CCR5 ZFN pair provides a durable source of modified cells and protects the CD4+ myeloid cells that are susceptible to HIV-1 as well.^140^ Further in vivo experiments showed that mice transplanted with ZFN-modified HSPCs experienced rapid selection for CCR5(-/-) cells, which had obviously lower levels of HIV-1 than the control group and maintained human cells throughout their tissues. The disruption of C-X-C chemokine receptor 4 (CXCR4) is also under exploration as a strategy for patients who harbor CXCR4-tropic HIV-1.^141^ Simultaneous genetic inactivation of both CCR5 and CXCR4 in human CD4+ T cells by ZFNs confers protection against viruses that exclusively use the targeted coreceptor.^142^ Nuclease platforms based on TALEN^143^ and CRISPR/Cas9^144–146^ are also being applied to disrupt CCR5 in T cells and HSPCs. Laboratory results from Ebina and Hu et al.^144,147^ showed that CRISPR/Cas9 not only could specifically eradicate latent HIV infection but also could prevent new HIV infection. Similarly, Hendel et al.^146^ recently demonstrated that the codelivery of chemically modified CCR5 sgRNA with Cas9 mRNA/protein enhanced the genome editing efficiency of human primary CD4+ T cells and CD34+ HSPCs, with no DNA delivery-associated toxicity.

The sustained expression of high-risk human papillomavirus (HPV) oncogenes E6 and E7 is implicated in malignant transformation and is strongly associated with cervical cancer.^148^ The targeted mutagenesis of those high-risk HPV genes by gene editing tools may be a potential genetic therapy and may reverse cervical cancer in situ. Ding et al.^149^ constructed a ZFN that could specifically recognize and cleave HPV16/18 E7 DNA. In their study, ZFN-mediated HPV16/18 E7 DNA disruption directly decreased the expression of E7, which resulted in efficient growth inhibition and type-specific apoptosis in HPV16/18-positive cervical cancer cells in vitro. When different plasmid-encoded zinc-finger modules were introduced in vivo, the therapeutic effects of ZFNs were further confirmed, inhibiting tumor growth in mice bearing cervical cancer cells. Similar results in another study showed that using ZFNs to target HPV E7 induced specific shear of the E7 gene and attenuated its malignant biological effect.^150^ Wayengera et al.^151^ computationally generated paired zinc-finger arrays (pZFAs) to target and cleave the genomic DNA of HPV-type 16 and 18, respectively. The authors highlighted the therapeutic effect of ZFN-mediated gene disruption in HPV 16/18, which was achieved when HPV-derived viral plasmids or vectors were introduced into precancerous lesions to realize targeted mutagenesis and gene-therapeutic reversal of cervical neoplasia. Additionally, the combined treatment of ZFNs with two chemotherapeutic drugs (cisplatin and trichostatin A) increased the apoptotic rate by approximately two times more than that of ZFNs used alone in HPV16/18-positive cervical cancer cells. Both chemotherapeutic drugs coordinated with ZFNs to downregulate HPV16/18 E7 expression while elevating retinoblastoma 1 (RB1) expression.^150^ TALEN-mediated targeting of HPV oncogenes E6 and E7 within host DNA resulted in restoration of the host tumor suppressors p53 and RB1, which not only reduced tumorigenicity in HPV-positive cell lines but also ameliorated HPV-related cervical malignancy in transgenic mouse models.^152^ Furthermore, CRISPR‐Cas9/HPV16 E6/E7 sensitized cervical cancer cells to cisplatin, indicating the potential of application in cervical cancer therapy.^153^

Hepatitis B virus (HBV) is the most important pathogen of liver disease. Cotransfection of engineered ZFN pairs with a target plasmid containing the HBV genome results in specific cleavage.^154^ Rananan et al.^155^ designed and screened an efficient gRNA targeting the HBV genomic locus and transmitted the sgRNA/Cas9 system by lentiviral vector to HepG2 cells that were integrated with HBV. Finally, the amount of covalently closed circular DNA (cccDNA) gradually decreased, dropping by 92% on the 36th day; HBV gene expression and replication were also inhibited. One study also attempted to knock out Epstein–Barr virus (EBV)-related genes using CRISPR/cas9 technology to treat latent infections caused by EBV.^156^ They used a plasmid containing CRISPR/cas9 to treat Raji cells isolated from Burkitt’s lymphoma with EBV latent infection; then, they found that cell proliferation was significantly inhibited and intracellular EBV load was significantly reduced.

Genomic editing technology allows us to gain a deeper understanding of the mechanisms underlying variant diseases associated with viral infection and demonstrates tremendous potential in the development of therapeutic approaches against viral infections, which represent some of the most intractable diseases.

### Hereditary eye diseases

In recent years, with the advancement of gene sequencing technology, it is more explicit to make the genetic diagnosis of a variety of hereditary eye diseases, such as congenital cataract, congenital glaucoma, retinitis pigmentosa (RP), congenital corneal dystrophy, Leber congenital amaurosis (LCA), retinoblastoma (RB), and Usher syndrome.^157^

CRISPR/Cas9 has already been used to generate animal models of RP. Receptor expression enhancer protein 6 (REEP6), a member of the REEP/Yop1 family of proteins, influences the structure of the endoplasmic reticulum.^158^ Arno et al. reported that biallelic mutations in REEP6 cause autosomal-recessive retinitis pigmentosa.^159^ They identified variants in REEP6 in patients with RP from unrelated families. Moreover, they created a knock-in mouse model of Reep6 p.Leu135Pro via CRISPR/Cas9. The clinical phenotypes of RP were replicated in the Reep6L135P/L135P homozygous knock-in mice, such as developing photoreceptor degeneration and dysfunction of the rod photoreceptors, which provides a better animal model for future studies of RP. The rodless (rd1) mouse, the most vastly used preclinical model of RP, has been aggressively debated for nearly a century after its occurrence because the cause of the blinding RP phenotype remains undetermined. The rd1 mouse has two homozygous variants in the Pde6b locus of chromosome 5: a nonsense mutation (Y347X) and a murine leukemia virus (Xmv-28) insertion in the reverse orientation in intron 1.^160,161^ Wu et al. repaired the nonsense point mutation via CRISPR/Cas9 to rescue and ameliorate the disease, demonstrating that the Y347X mutation in rd1 mice is pathogenic.^162^ Another animal model of RP, the transgenic S334ter-3 rat, possesses the mutation RhoS334, which shows similar phenotypes to human class I RHO mistracking mutations, leading to a continual degeneration of photoreceptors and vision decline.^163,164^ The protospacer adjacent motif (PAM) sequence in RhoS334 (5′-TGG-3′) diverges from the PAM in RhoWT (5′-TGC-3′) by only one nucleotide. Benjamin et al. reported that an allele-specific disruption of RhoS334 via a single subretinal injection of CRISPR/Cas9 and gRNA by electroporation prevented retinal degeneration and increased visual acuity.^165^ Additionally, Latella et al. successfully edited the human rhodopsin (RHO) gene by the electroporation of plasmid-based CRISPR/Cas9 in a P23H transgenic mouse model for autosomal dominant RP and confirmed its efficacy as a genetic engineering tool in photoreceptor cells,^166^ which strongly demonstrates that the CRISPR/Cas9 system is an efficient and promising therapeutic tool for retinal degeneration, such as RP. Suzuki et al. also determined a CRISPR/Cas9-mediated homology-independent targeted integration (HITI) strategy and demonstrated its efficacy in ameliorating visual function in a rat model of RP.^167^ HITI is a targeted integration mediated by NHEJ, and this study is the first time that HITI could play a role in nonmitotic cells. The advantage of HITI technology is that it can be applied to any targeted genome engineering system, not just CRISPR/Cas9.

The combination of CRISPR/Cas9 technology and other methods provides new avenues for the treatment of related eye diseases, such as treatment with AAV and iPSCs. Bassuk et al. first reported that CRISPR/Cas9 precisely repairs retinitis pigmentosa GTPase regulator (RPGF) point mutations, which cause X-linked RP in patient-specific iPSCs; this supports that combining gene editing with autologous iPSCs could be a personalized iPSC transplantation strategy for therapies of various retinal degenerations.^168^ Similarly, Deng et al. found that iPSC-derived retinal organoids from three RP patients with different frameshift mutations in the RPGR gene have significant defects in photoreceptors, including defects in their morphology, localization, and electrophysiological activity. The correction of an RPGR mutation via CRISPR/Cas9 reverses ciliopathy and rescues photoreceptor loss, which indicates that CRISPR/Cas9 can serve as an adopted mutation repair strategy.^169^

LCA is a congenital retinal dystrophy that causes significant vision loss at an early age.^170^ To verify that mutation in human KCNJ13 causes LCA, Zhong et al. employed CRISPR/Cas9 to create Kcnj13 mutant mice by zygote injection with sgRNA and spCas9 mRNA. Kcnj13 mutant mice showed a declined response to light, a loss of photoreceptors and rhodopsin mislocalization, revealing that the loss of Kcnj13 function could mimic human LCA phenotypes in mice.^171^ As demonstrated by Zhong et al., CRISPR/Cas9 could accelerate the study of candidate gene function in biology and disease.^171^ The centrosomal protein 290 kDa (CEP290) gene, the most frequent mutation in LCA, causes the most common subtype of LCA, which is referred to as LCA10. However, the large size of CEP290 exceeding the capacity of AAV delivery prevents the use of this delivery platform. To overcome this capacity limitation, Ruan et al. used dual recombinant AAV vectors to induce the CRISPR/Cas9-mediated deletion of a specific intronic fragment of the Cep290 gene in mouse photoreceptors.^172^ Additionally, using a smaller *S. aureus* CRISPR/Cas9 system enables a single AAV vector to deliver the Cas9 gene and two gRNAs, which performs a dual-cut excision of the CEP290 mutation-containing region in primary fibroblasts from LCA10 patients.^164^ Recently, Maeder et al. developed a candidate genome editing therapy named EDIT-101 to restore vision loss in LCA10.^173^ They delivered the *Staphylococcus aureus* Cas9 and CEP290 gRNA to the photoreceptor via an AAV5 vector. Humanized CEP290 mice showed rapid and continuing CEP290 gene editing after subretinal delivery of EDIT-101. These extraordinary studies provide a roadmap for the preclinical advance of gene therapy for LCA10.

RB is the most common pediatric eye tumor of the developing retina.^174^ Approximately one-third of RB cases are caused by biallelic RB1 mutation or deletion. Solin SL et al. reported that using TALEN gene editing to inactivate somatic rb1 in adult zebrafish induced tumorigenesis at high frequency.^175^ A highly penetrant and rapid RB preclinical model was reported by Naert et al., utilizing the CRISPR/Cas9 system to induce the knockout of rb1 and retinoblastoma-like 1 (rbl1) in *Xenopus tropicalis*.^176^ The animal model showed rapid development of RB, and it will be a good model for early stage drug discovery and rapid therapeutic target identification. Jian Tu et al. generated a pluripotent H1 human embryonic stem cell line with RB1 heterozygous knockout by CRISPR/Cas9 nickase, which provides a valuable cell resource for the study of hereditary retinoblastoma.^177^ Glaucoma is the second leading cause of blindness worldwide and is characterized by elevated intraocular pressure (IOP).^178^ Gain-of-function mutations in myocilin (MYOC) have been reported to commonly cause primary open-angle glaucoma (POAG).^179–181^ The accumulation of mutated myocilin inside cells leads to the activation of the unfolded protein response (UPR) cascade and endoplasmic reticulum (ER) stress in the trabecular meshwork (TM).^182,183^ TM cells are sensitive to chronic ER stress and finally die, resulting in increased IOP and glaucoma.^184,185^ Jain et al. knocked down the expression of mutant MYOC in a mouse model of POAG by CRISPR/Cas9, resulting in the reduction of ER stress, lower IOP, and the preventability of further glaucomatous damage in mouse eyes.^186^ Importantly, they also demonstrated the feasibility of utilizing CRISPR/Cas9 in human eyes with glaucoma. A dominant-negative mutation in KRT12,^187^ which causes Meesmann epithelial corneal dystrophy (MECD), results in the occurrence of a novel *Streptococcus pyogenes* PAM. Courtney et al. designed a sgRNA complementary to the sequence adjacent to this PAM and found that this sgRNA has a large effect on the decrease in mRNA and protein of KRT12 in vitro.^188^ The injection of combined Cas9/sgRNA into the corneal stroma of a humanized MECE mouse model showed frame-shifting deletions of the mutated KRT12 allele. This study is the first to demonstrate the in vivo allele-specific CRISPR/Cas9 gene editing of a novel PAM created by a heterozygous disease-causing SNP.^188^

### Hematological diseases

Nearly half of hemophilia A cases are caused by the inaccurate expression of factor VIII (F VIII) due to inversion of the chromosome.^189^ In one study, iPSCs were derived from somatic cells of hemophilia A patients induced by chromosome inversion, and the F VIII gene of iPSCs was modified by CRISPR/Cas9 technology.^190^ The modified iPSCs were induced to differentiate into mature endothelial cells capable of expressing factor VIII and then transplanted into hemophilia mice lacking factor VIII. The results showed that the transplanted mice began to produce factor VIII, which effectively inhibited bleeding symptoms. Hemophilia B is caused by a deficiency in factor IX (F IX). Coagulation activity can be restored by increasing FIX in plasma. Guan et al.^191^ found that the F9 gene carries a new mutation, Y371D, in a family of hemophilia B patients, which leads to a more severe hemophilia B phenotype than the previously discovered Y371S mutation. They used naked DNA constructs and adenoviral vectors to deliver Cas9 to adult F9 Y371D mutant mice. After treatment, it was found that when adenovirus was used as a vector to deliver cas9, although the mutation gene was highly efficiently repaired, hepatotoxicity was severe. However, Cas9 with a naked DNA structure successfully repaired more than 0.56% of F9 alleles in hepatocytes in hemophilia B mice, enough to restore hemostasis. CRISPR technology also provides a quick path to build hemophilia models. Researchers from the Institute of Zoology in the Chinese Academy of Sciences injected the CRISPR/Cas9 system targeting vwF (vascular hemophilia mutant gene) into the fertilized eggs of miniature pigs and obtained the double allele mutant mini-pig quickly and efficiently. These miniature pigs have severe coagulopathy, indicating the successful construction of a miniature pig model of von Willebrand disease by CRISPR technology.^192^

Sickle anemia is the first genetic disease with a clearly understood pathogenesis. A single nucleotide mutation from A to T in the first exon of human β-globin results in a lesion.^193^ In 2016, a Stanford University team reported on the use of CRISPR/Cas9 technology to repair β-globin gene (HBB) mutations in patient-derived HSCs in vitro.^194^ After the modified iPSCs differentiated into red blood cells, normal HBB mRNA could be detected. This preclinical experiment provided theoretical support for gene editing technology in the treatment of sickle anemia. The CRISPR/Cas9 system has also been used to correct β thalassemia-causing mutations in the HBB.^195^ Using CRISPR/Cas9 technology to direct the calibrated DNA sequence to the HBB mutation site, it was possible to correct two different β-thalassemia mutations in the HBB gene of patient iPSCs by HR.

### Other hereditary diseases

Duchenne muscular dystrophy (DMD) is the most common form of muscular dystrophy caused by mutations of the DMD gene.^196^ Current X-linked muscular dystrophy (mdx) mice can only partially mimic human disease conditions. Their small size, limited chronic muscle damage and muscle weakness also impose limitations on disease research and analysis. Therefore, larger animals such as rats, rabbits or pigs are more valuable for preclinical studies. Larcher et al.^197^ generated Dmd^mdx^ rats by targeting exon 23 of DMD with TALENs. These edited rats showed a significant reduction in muscle strength and decreased spontaneous motor activity. Sui’s team generated DMD knockout rabbits by coinjecting Cas9 mRNA and sgRNA into rabbit zygotes targeting exon 51 of DMD. These rabbits harbored the typical phenotypes of DMD, and the pathological features in the diaphragm and heart were similar to those of DMD patients.^198^ In addition, the monkey dystrophin gene was targeted using CRISPR/Cas9 to create mutations that cause DMD. The detection of the relative targeting rate showed that CRISPR/Cas9 could result in mosaic mutations in up to 87% of the dystrophin alleles in monkey muscle.^199,200^ Notably, three groups of researchers have recently described the use of CRISPR/Cas9 to remove mutations in the DMD gene encoding dystrophin, which affects protein expression.^201–203^ The investigators used the CRISPR/Cas9 system to excise the mutant portion of DMD in the mdx mouse model, thereby synthesizing a shorter version of dystrophin protein in the muscle fibers and restoring partial muscle function. This provided a promising method for correcting disease-causing mutations in the muscle tissue of patients.

Patients with primary immunodeficiencies lack a part of their immune system or have immune system dysfunction, and they can be treated with allogeneic HSC transplantation.^204^ This may be a high-risk process when leukocyte antigen-matched donors lack tissue compatibility. Correcting a patient’s own HSCs through gene therapy provides an attractive option. HSCs can also be used in situ to correct pathogenic mutations and to develop cell or animal models to study the pathogenic effects of specific genetic defects found in immunodeficient patients. As the most severe immunodeficiency, severe combined immunodeficiency (SCID) is caused by a mutation in the gene encoding the interleukin 2 receptor gamma (IL2RG), which results in the developmental arrest of T cell production and additional primary or secondary defects in B cells. Several research teams have successfully used ZFN and TALEN techniques to induce HDR at the IL2RG locus in various human cell types, including HSCs and embryonic stem cells (ESCs).^205–207^ Other studies have utilized endonucleases to generate different kinds of immunodeficient animal models that were previously unable to be established due to a lack of effective genetic modification.^207–210^ As a result of engineered nuclease-mediated editing of genomic modifications, other animal disease models have been developed, simulating Rett syndrome,^211^ hereditary deafness,^212^ Wilson disease,^213^ Laron syndrome,^214^ Niemann–Pick disease,^215^ Netherton syndrome,^216^ and so on. Advances in genome editing technologies will further expand the application of animal models in disease mechanism research and treatment development.

## Future application prospects

### Genome editing in cancer immunotherapy

**Fig. 3:**
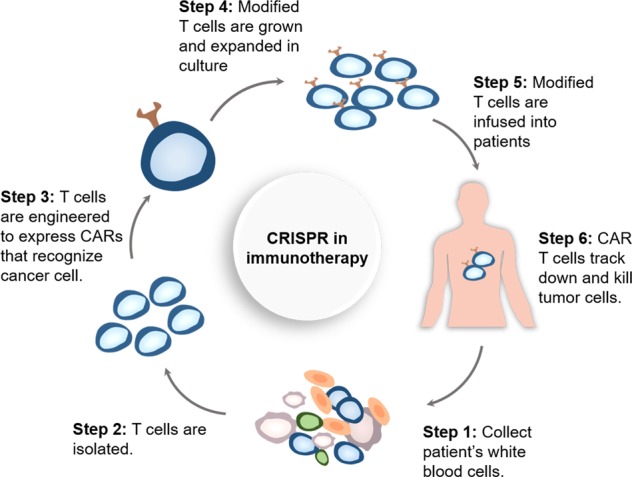
Production of CAR T cell products with genome editing technology.

Recently, cancer immunotherapy has stimulated great interest, with its goal to harness the patient’s own immune system against tumor cells.^217^ One promising area in immunotherapy is the application of genetically engineered T cells, known as chimeric antigen receptor (CAR) T cells, which allow the targeting of tumor-associated antigens and could enhance the therapy response.^218,219^ The preparation of functional CAR T cells requires several key steps (Fig. 3): first, the patient’s white blood cells are collected, and the patient’s T cells are isolated via leukapheresis, after which T cells are reengineered and modified with tumor-antigen-specific receptors and costimulating molecules; next, a CAR-containing viral vector is transduced into the modified T cells, followed by the amplification of the CAR-expressing T cells and then infusion of the cells into the patient. CARs are synthetic receptors that typically contain the following parts: an antibody-derived targeting ectodomain that recognizes tumor antigens; a costimulatory molecule region that can bind to receptors such as CD28, 4-1BB, or CD278;^220^ and a T cell signaling domain. After binding to a particular antigen, the CAR can transmit signals and activate modified T cells. The independence of CAR recognition endows genetically engineered CAR T cells with a fundamental antitumor advantage by avoiding the limitation traditionally conferred by the major histocompatibility complex (MHC).^221^ However, due to the complexity of the manufacturing process, the limited selection of target antigens and the insufficient antitumor responses to solid tumors, the applicability of this transformative product is highly limited. Over the past few years, flexible gene editing technologies have become significant engineering tools to address these limitations and further improve CAR T designs.

The development of allogeneic CAR T cell therapy would simplify and solve some challenges in the process of manufacturing autologous CAR T cells.^222^ The endogenous αβ T cell receptor (TCR) is responsible for major and minor histocompatibility antigen recognition. By genetically disrupting various parts of the αβ TCR complex and/or the human leukocyte antigen (HLA) class I loci of allogeneic T cells, it is possible to create a universal cellular therapy product that confers a wider range of application capability with minimal related adverse effects, including graft-versus-host disease (GVHD). In 2012, Torikai et al. used engineered ZFNs to eliminate the expression of α or β chains in endogenous TCRs, leading to the loss of TCR function in CD19 CAR T-cells.^223^ These modified T cells did not respond to TCR-specific stimuli but retained the ability to recognize and target CD19, leading to the generation of universal allogeneic tumor-associated antigen-specific CAR T cells. With the same approach, the selective elimination of HLA expression was achieved in CD19-specific T cells and in embryonic stem cells, which increased the applicability of this strategy by avoiding the infusion of HLA-disparate immune cells.^224^ Similar work was also performed by Poirot et al. using TALEN-mediated editing in 2015. By the application of TALEN-mediated gene editing, the expression of αβ TCR was inactivated, eliminating the possibility of T cell responses to allogeneic antigens and GVHD.^74^ The beneficial role of TCR-depleted CD19 CAR T cells in evading GVHD has recently been validated in two infant patients with relapsed refractory CD19+ B cell acute lymphoblastic leukemia, leading to successful molecular remissions within 4 weeks.^225^ In addition, the target of the lymphocytic depleting monoclonal antibody alemtuzumab, CD52, a human glycoprotein found on the surface of lymphocytes, was simultaneously disrupted by TALENs to eliminate the potential of any remaining alloreactive T cells and to promote the engraftment of cellular therapies. As a proof of application of this platform, TCR/CD52-deficient CAR T cells were administered concurrently with alemtuzumab and demonstrated antitumor activity in a lymphoma murine model similar to unmodified anti-CD19 CAR T cells, with resistance to alemtuzumab destruction.^226^

The widespread use of gene editing techniques based on ZFNs and TALENs has been hampered by the requirement to design specific nuclease pairs for each new gene target. The development of the CRISPR/Cas9 system has successfully promoted multiple gene editing in CAR T cells in a faster and easier way. Using this technology, Liu et al. efficiently generated CAR T cells in which two (TRAC and B2M) or three genes (TRAC, B2M and PD-1) were simultaneously disrupted and tested their antitumor function in vitro and in vivo.^227^ To target the first exon of TRAC and B2M, they designed four sgRNAs. To target the first exon of PD-1, two sgRNAs were designed, and one published sgRNA was tested. Finally, double-knockout (B2M and TRAC) T cells were induced with high efficiency, yet in triple-knockout (B2M, TRAC and PD-1) T cells, only 64.7% of the clones of the PD-1 PCR products were mutants, which implies that PD-1 expression might be downregulated during T cell expansion. More importantly, the CRISPR/Cas9-mediated multiplex gene-edited CAR T cells maintained CD19-specific antitumor function in a lymphoma xenograft mouse model, suggesting that they are promising reagents for cancer treatment. In another interesting study,^228^ the efficient double knockout of endogenous TCR and HLA class I molecules was achieved by a one-shot CRISPR protocol that incorporated multiple gRNAs into a CAR lentiviral vector to generate allogeneic universal CAR T cells. In this study, CRISPR/Cas9 mediated the simultaneous knockout of four loci of the T cell surface receptors PD-1 and CTLA-4 and successfully generated allogeneic universal T cells. More recently, the CRISPR/Cas9-mediated generation of CAR T cells that specifically disrupt inhibitory immune receptors such as T cell membrane protein-3 (TIM-3),^229^ adenosine 2a receptor (A2aR)^230^ and lymphocyte-activation protein 3 (LAG-3)^231^ have shown a better percentage of complete remission in xenograft mouse models by increasing the secretion of antitumor-related cytokines (such as IFN-g, GM-CSF and MIP-1b). These factors may be involved in CAR T cell exhaustion and acute myeloid leukemia (AML) dysfunction, as the combination of checkpoint inhibitors with CAR T cells may result in the enhanced antitumor efficacy of AML and other hematological malignancies.

Taken together, these results suggest that genome editing could serve as a good platform for generating “universal” CAR T cells and can be applied to the large-scale production of healthy “off-the-shelf” T cells against multiple targets.

### Screening for functional genes

The concept of precision medicine has led to the development of many targeted drugs for the treatment of different diseases. For example, targeted drugs designed for known carcinogenic sites will specifically bind to carcinogenic components (gene fragment or protein) and induce the apoptosis of tumor cells without affecting normal tissue cells. However, one obvious drawback of this molecular targeting therapy is that a certain mutation or gene expression alteration is necessary for patients to respond to the targeted drug; otherwise, drug resistance persists. Based on CRISPR/Cas9 technology, scientists have established mammalian genome-wide mutation libraries or libraries of gene mutations associated with certain functions, which are related to screening phenotypes through functional screening and subsequent PCR amplification and deep sequencing analysis. The entire process is called the CRISPR/Cas9 gRNA library screening technology.^232,233^ The gRNA library is an ideal tool for drug screening or the targeted screening of specific pathways. The establishment of gRNA libraries will play an important role in functional gene screening, disease mechanism research and drug development. Functional genome screening using the CRISPR system could reveal changes in gene expression after cancer drug therapy and help to investigate drug-gene interactions by adding small molecules as perturbations, thereby identifying novel targets for precise treatment and providing insights into disease development.^234,235^

One of the chief goals of pooled CRISPR/Cas9 unbiased screening in cancer research is to identify genotype-specific vulnerabilities, and AML was the first disease to be systematically analyzed with this technology.^236^ Using this platform, the authors found several well-known potential targets for AML therapies, including BCL2, BRD4, MEN1, and DOT1L, by studying five commonly used AML cell lines and two solid tumor cell lines as controls. Since then, large-scale CRISPR/Cas9 screening has been performed to systematically discover essential genes in many cancer cell lines^237,238^, and approximately 1500 essential genes have been identified, which is five times higher than the number of genes previously detected by shRNA screening.^239^ Another successful example involved the use of CRISPR/Cas9-mediated loss-of-function screening to identify cancer metastasis-related genes.^240^ In this study, a nonmetastatic lung cancer cell line was infected with the mouse genome-scale CRISPR knockout (mGeCKO) sgRNA library and subcutaneously transplanted into immunocompromised mice. After 6 weeks, enriched sgRNA sequencing was performed in mice with lung cancer metastasis, and several candidate genes related to lung metastasis were identified and verified, including the already known genes PTEN^241^, miR-345,^242^ and miR-152^243^ and several new genes, including Fga, Trim72 and Nf2. With a CRISPR-based strategy, another loss-of-function screening identified four candidate HCC suppressor genes that had not previously been associated with HCC (Nf1, Plxnb1, Flrt2, and B9d1). The authors also found that these suppressor genes were closely related to the RAS signaling pathway through the intervention of small molecule inhibitors.^244^ A CRISPR-based double-knockout (CDKO) system has also been developed in K562 leukemia cells. The system uses dual sgRNA libraries to screen for combinatorial genes and identify pairs of synthetic lethal drug targets.^245^ Recent landmark studies have demonstrated the power of CRISPR/Cas9 to discover long noncoding (lncRNA) loci. These studies applied CRISPR-interference (CRISPRi)- or CRISPR-activation (CRISPRa)-based libraries to screen for functional lncRNA loci that could modify cell proliferation^246,247^ and drug resistance^235,248^. Generally, a comprehensive sgRNA library was designed to target the initiation site of lncRNA transcription, and then the library was transduced into different cell lines. Then, through sequence analysis, hundreds to thousands of lncRNAs promoting cell growth and drug resistance could be identified.

Depending on each mutation’s individual effect, the simultaneous mutation of two genes can produce an unexpected phenotype that determines the potential functional relationship between genes.^249^ This phenomenon, known as genetic interaction, has implications for the development of cancer therapeutics; for example, in cancers with loss-of-function mutations in BRCA1 or BRCA2, an inhibitor of PARP1/2 (e.g., olaparib) could result in cell killing by simultaneously disrupting both genes.^250^ The CRISPR/Cas9 system provides an effective strategy for identifying synergistic gene interactions to gain insights into the response of cancer to chemotherapy. A CRISPR-based double-knockout system combined with deep sequencing, phenotypic measurement and genetic analysis has identified interactions between the synergistic drug targets in K562 leukemia cells, such as BCL2L1 and MCL1.^245^ Similarly, the double-knockout screening method was used to detect 73 tumor genes in pairs and found synthetic lethal interactions of many known (e.g., BRCA-PARP) and unknown genes, approximately 75% of which could be replicated using combinatorial drugs.^251^ Combining pooled CRISPR screening with a perturbed drug could identify genes that synergize or confer resistance to the agent.^252^ In one of the first pooled CRISPR screens, the BRAF inhibitor vemurafenib was used to treat a genome-scale knockout library of melanoma cells and recovered genes conferring resistance to the drug.^253^ Similar to the CDKO system, another simple and effective strategy for analyzing the function of combinatorial genes is CombiGEM-CRISPR (combinatorial genetics en masse-CRISPR).^232^ It combines two pooled sgRNA libraries in one vector, and some genetic hits (such as KDM6B and BRD4) were discovered by this method. Disrupting these genes with the CombiGEM system demonstrated a stronger synergistic effect on the proliferation of tumor cells compared to previously reported small molecule inhibitors. Likewise, a series of CRISPR-based screening techniques has been performed to identify genes that regulate cellular response to specific drugs, such as TRAIL,^254^ ATR,^255^ or Ras^256^ pathway inhibitors. Of note, an in vivo screening based on CRISPR/Cas9 has identified protein tyrosine phosphatase nonreceptor type 2 (PTPN2) as a novel target for cancer immunotherapy.^257^ In the future, this innovative approach could also be used to develop personalized cancer therapies based on genotype-specific targets.^258^

### Gene diagnostic tools

Cancer predisposition genes describe genes in which germline mutations result in an increased risk of cancer.^259^ Identifying such sensitive genes through genetic diagnosis is critical for cancer prevention. However, low-frequency mutations are not easily identified by sequencing, and a CRISPR-based diagnostic system referred to as SHERLOCK (specific high sensitivity enzymatic reporter UnLOCKing) has been established to solve this problem.^260^ Technically, the system consists of two important elements, the RNA-guided endonuclease Cas13a (another Cas family member) and the reporter signal. Cas13a exists as a key factor and effectively induces trans-cleavage of nonspecific single-stranded DNA (ssDNA). The reporter signal is released after RNA cleavage. This approach appeared to be a highly sensitive detection method when used to detect two cancer mutants, BRAF V600E and EGFR L858R.^57^ Another system called DETECTR (DNA endonuclease-targeted CRISPR trans-reporter) has also been developed.^261^ Cas12a acts like Cas13a in this system, and another enzyme, recombinase polymerase amplification (RPA), is used as a detection tool to screen for viral infections in cancer and to amplify microsamples. The system seemed to be a fast and inexpensive method for detecting HPV 16/18 in lung carcinomas.^262^ In the study of breast cancer, the CRISPR nuclease-dead Cas9 (dCas9) system was fused to a DNA methyltransferase effector and infected healthy breast cells by lentivirus. Through this technology, researchers have discovered that the cyclin-dependent kinase inhibitor 2A (CDKN2A) gene was a key driver in carcinogenesis, which led to abnormally rapid cell division and might become an early diagnostic marker for breast cancer.^263^ Additionally, CRISPR/Cas9 gene editing as well as overexpression experiments have also confirmed that the BRCA1-delta11q optional splice isoform is a primary factor in PARPi and cisplatin treatment resistance in breast cancer.^264^

## Application of gene editing in clinical trials

Genome editing, as an attractive and challenging therapeutic approach, can correct or eliminate mutations that lead to the development of cancer and other genetically driven diseases. So far, ex vivo genome editing has been the most widely used, that is, the genetic engineering of cells in vitro and then the modified cells are re-engrafted back to patients. In recent years, teams represented by China and the United States have conducted a series of clinical trials of gene editing, such as producing more effective CAR T cells for the treatment of cancer and the knockout of the erythroid specific enhancer of BCL11A to upregulate gamma globulin in autologous erythroid HSCs as a potential therapy for sickle cell disease and β-thalassemia (Table 2).

**Table 2:** Clinical trials of gene editing in the treatment of human diseases.

Platform	Disease applications	Target	Edited cells	Delivery	Sample size	Phase	Trial number
ZFN	HIV-1 infection	CCR5	CD4+ T cells	Adenovirus	12	I	NCT00842634
	HIV-1 infection	CCR5	CD4+ T cells	Adenovirus	19	I	NCT01044654
	HIV-1 infection	CCR5	CD4+ T cells	Adenovirus	21	I/II	NCT01252641
	HIV-1 infection	CCR5	CD4/CD8 T cells	Adenovirus	26	I	NCT01543152
	HIV-1 infection	CCR5	CD4/CD8 T cells	mRNA	12	I/II	NCT02225665
	HIV-1 infection	CCR5	CD4+ T cells	mRNA	14	I	NCT02388594
	HIV-1 infection	CCR5	CD4+ T cells	mRNA	30	I/II	NCT03666871
	HIV-1 infection	CCR5	CD4+ T cells	mRNA	12	I	NCT03617198
	HIV-1 infection	CCR5	CD34 + HSPCs	mRNA	18	I	NCT02500849
	HPV-induced cervical precancerous lesions	HPV16/18 E7	Epithelial cells	DNA	20	I	NCT02800369
	Mucopolysaccharidosis I	IDS gene	Hepatocytes	AAV	9	I/II	NCT03041324
	Mucopolysaccharidosis II	IDUA gene	Hepatocytes	AAV	3	I/II	NCT02702115
	Hemophilia B	Factor IX gene	Hepatocytes	AAV	12	I	NCT02695160
	β-Thalassemia	BCL11A gene	CD34 + HSPCs	mRNA	6	I/II	NCT03432364
	Recurrent/refractory malignant glioma	IL13Ralpha2	CD8 + T cell	Injection	6	I	NCT01082926
TALEN	Relapsed/refractory B-ALL	CD52, TRAC	CAR T cells	Lentivirus	18	I	NCT02808442
	HPV-related cervical intraepithelial neoplasia	HPV16/18 E6/E7	Epithelial cells	Plasmid	40	I	NCT03226470
TALENs and CRISPR/Cas9	HPV-related cervical intraepithelial neoplasia	HPV16/18 E6/E7	Epithelial cells	Plasmid	60	I	NCT03057912
CRISPR/Cas9	AML	CD123, TRAC	CAR T cells	mRNA	162	I	NCT03190278
	Metastatic non-small cell lung cancer	PDCD1	T cells	DNA	12	I	NCT02793856
	Castration-resistant prostate cancer	PDCD1	T cells	DNA	Withdrawn	I	NCT02867345
	Muscle-invasive bladder cancer	PDCD1	T cells	DNA	Withdrawn	I	NCT02863913
	Advanced esophageal cancer	PDCD1	T cells	DNA	16	I	NCT03081715
	Metastatic renal cell carcinoma	PDCD1	T cells	DNA	Withdrawn	I	NCT02867332
	HIV-1 infection with ALL	CCR5	CD34+ HSPCs	Liposome and electroporation	5	I	NCT03164135
	EBV-positive cancers	PDCD1	T cells	DNA	20	I	NCT03044743
	Relapsed refractory multiple myeloma, melanoma, synovial sarcoma, and myxoid/round cell liposarcoma	NY-ESO-1, TRAC PDCD1	T cells	Lentiviral and electroporation	18	I	NCT03399448
	Relapsed or refractory CD19+ leukemia and lymphoma	TRAC, B2M	CAR T cells	Lentiviral and electroporation	80	I/II	NCT03166878
	Relapsed or refractory CD19- leukemia and lymphoma	CD19 and CD20 or CD22, TRAC	CAR T cells	Lentiviral and electroporation	80	I/II	NCT03398967
	Mesothelin-positive multiple solid tumors	PDCD1 and TRAC	CAR T cells	Lentiviral DNA	10	I	NCT03545815
	Mesothelin-positive multiple solid tumors	PDCD1 and TRAC	CAR T cells	Lentiviral DNA	10	I	NCT03747965
	Metastatic gastrointestinal epithelial cancer	CISH	TIL	Electroporation	Withdrawn	I/II	NCT03538613
	T cell leukemia or lymphoma	CD7, CD28	CAR T cells	–	21	I	NCT03690011
	Neurofibromatosis type 1	NF1	iPSCs	DNA	20	I	NCT03332030
	β-Thalassemia	HBB gene	iHSCs	–	12	I	NCT03728322
	β-Thalassemia	BCL11A gene	CD34+ HSPCs	–	45	I/II	NCT03655678
	Sickle cell disease	BCL11A gene	CD34 + HSPCs	–	45	I/II	NCT03745287
	LCA10	CEP290 gene	Photoreceptor cells	AAV	18	I/II	NCT03872479

*ZFN* zinc-finger nuclease, *CCR5* chemokine receptor 5, *HSPCs* hematopoietic stem/progenitor cells, *IDS* iduronate 2-sulfatase, *IDUA* α-L-iduronidase, *BCL11A* mouse B cell lymphoma factor 11A, *B-ALL* B acute lymphoblastic leukemia, *TRAC* T cell receptor alpha chain, *TALEN* transcription activator-like effector nuclease, *CRISPR* clustered regularly interspaced short palindromic repeat, *AML* acute myeloid leukemia, *PDCD1* programmed cell death 1, *NF1* neurofibromatosis type 1, *TIL* tumor-infiltrating lymphocytes, *iPSCs* induced progenitor stem cells, *iHSCs* induced hematopoietic stem cells, *LCA10* Leber congenital amaurosis type 10, *CEP290* centrosomal protein 290, *AAV* adeno-associated virus

### Anticancer clinical trials

The gene editing clinical trial using the ZFN product GRm13Z40-2 for the treatment of stage III or IV malignant glioma patients (NCT01082926) was launched in 2010. The ZFN-mediated GRm13Z40-2, an allogeneic CD8+ cytolytic T cell line genetically modified to express the glucocorticoid-resistant IL13-zetakine, was delivered to tumor cells by intratumoral injection. In another phase I clinical trial (NCT02800369), ZFN agents (ZFN-603 and ZFN-758) were transfected into HPV-infected cervical epithelial cells to determine whether these agents could block the malignant progression of cervical intraepithelial neoplasia and reduce the incidence of cervical cancer. To date, this study has finished the data collection phase. Only two studies using TALENs in CAR T cells have been reported. One study (NCT02808442) developed a portfolio of allogeneic, universal CAR T cells (UCART19) that target relapsed or refractory CD19-positive B-acute lymphoblastic leukemia. In this study, alloreactivity and alemtuzumab sensitivity were eliminated by disrupting the loci encoding TRAC and CD52. A similar concept is used to generate allogeneic TALEN-edited CAR T cells that target CD123 (UCART123) in AMLs and blastic plasmacytoid dendritic cell neoplasms (NCT03190278).

Due to the simple design process and the ability to make multiple gene edits at one time, the CRISPR/Cas9 system has become an important tool in the development of cancer therapy. To date, eleven clinical trials have been carried out to assess the effectiveness of the CRISPR system in cancer therapy, seven of which are immunotherapies that target PD-1 protein expression. The first clinical trial using the revolutionary CRISPR/Cas9 technique for cancer treatment recruited its first patient in West China Hospital, Sichuan University in 2016.^265^ In this nonrandomized, open-label phase I study (NCT02793856), the safety of ex vivo engineered PD-1 knockout T cells has been evaluated in the treatment for metastatic non-small cell lung cancer with progression after all standard treatments. In this trial, PD-1 expression was disabled by CRISPR/Cas9 in peripheral blood lymphocytes harvested from the enrolled patients. The edited lymphocytes were isolated, expanded and subsequently reinfused into the patients. Ongoing clinical trials apply the same concept of PD-1 knockout autologous T cells to treat other cancer types, including prostate cancer (NCT02867345), esophageal cancer (NCT03081715) and renal cell cancer (NCT02867332). These trials can be considered as the first proof-of-concept studies to apply the in vitro CRISPR/Cas9 gene knockout technique in cancer therapy. There are now studies combining PD-1 knockout with other targeted editing in therapy development, which may lead to improved efficacy for clinical application. One example is the addition of PD-1 knockout to Epstein–Barr virus (EBV)-specific autologous T cells for the treatment of EBV-positive cancers, which is currently in phase I/II clinical trials (NCT03044743).

The elimination of endogenous TCR and PD-1 by CRISPR might enhance tumor rejection activity. Recently, the Recombinant DNA Advisory Committee (RAC) of the US National Institutes of Health (NIH) approved a clinical trial to be piloted at the University of Pennsylvania. In this trial, PD-1 and the endogenous TCR will be abolished by CRISPR/Cas9 in HLA-A*0201 restricted NY-ESO-1 TCR redirected autologous T cells. Such redirected engineered T cells will be applied to a variety of cancer types, including relapsed refractory multiple myeloma, melanoma, synovial sarcoma, and myxoid/round cell liposarcoma (NCT03399448).

The use of CRISPR/Cas9 technology to generate CAR T cells to attack malignant cells has become a research hotspot in clinical trials. A clinical phase I/II trial (NCT 03166878) was conducted to evaluate the safety and tolerance of patients with recurrent or refractory CD19+ leukemia and lymphoma to several doses of universal CD19-specific CAR T cells (UCART 019). In this study, UCART019 cells were obtained by combining lentiviral delivery of CAR receptors and CRISPR RNA electroporation to simultaneously disrupt endogenous TCR and B2M genes. These cells are derived from one or more healthy unrelated donors but might help to avoid graft-versus-host-disease (GVHD) and reduce host-mediated immunity, thereby providing patients with anti-leukemic effects in a relatively safe condition. Unfortunately, a small number of patients relapsed due to the lack of CD19 expression in tumor cells. Therefore, another clinical trial (NCT03398967) that is more applicable for a wide range of patients focused on allogenic CRISPR-edited bispecific CD19+CD20+ or CD19+CD22+ CAR T cells, which could recognize and kill the CD19-negative malignant cells through the recognition of CD20 or CD22. In another study, a new clinical trial (NCT03057912) has proposed to evaluate the safety and efficacy of combination genome editing of TALENs and CRISPR/Cas9 by targeting HPV16 and HPV18 E6/E7 DNA in the treatment of HPV-associated cervical intraepithelial neoplasia. In this trial, CAR T cells edited by both techniques were administered twice a week for 4 weeks to disrupt target gene expression and promised to reduce off-target effects.

The mutation rate of the neurofibromatosis type 1 (NF1) gene is one of the highest in the human genome, which is likely to cause various benign or malignant tumors.^266^ In one trial (NCT03332030), CRISPR/Cas9 technology was designed to screen and identify NF1-specific drugs. First, a human iPSC library was established from NF1 patients with good phenotypic characteristics, and different cell lines (NF1+/+, NF1+/− and NF1−/−) were developed using CRISPR/Cas9. Then, potential therapeutic agents could be identified by examining the reversal or remission phenotypes after specific drug use. Although results from clinical trials in genome editing appear to be promising, more work needs to be done to ensure the safety and effectiveness of this tool in treating human cancers.

### Antiviral clinical trials

CCR5 acts as a major coreceptor in the early stage of HIV infection, and CXCR4 plays an important role as an auxiliary receptor when establishing stable infections. Treatment strategies targeting both coreceptors may avoid protection failure because coreceptor usage of HIV infection can be switched between CCR5 and CXCR4.^267^ The production of engineered immune cells resistant to HIV infection or replication is the primary strategy for genome editing-based HIV treatment. The most common method involves two steps: modifying the cells (CD4+ T cells and CD34+ hematopoietic stem/progenitor cells) in vitro and then reinfusing the modified cells into patients.^268,269^ Several clinical trials involving CD4+ T cell modification in the context of HIV infection have already been tested. The first approved genome editing trial involving the treatment of HIV with ZFNs (NCT00842634) began in 2009 to evaluate the safety and anti-HIV effects of modified autologous CD4+ T cells in HIV-1 infected patients. The ZFNs were delivered ex vivo to autologous CD4+ T cells by adenoviral vectors for CCR5 gene knockout, and each participant received a single infusion of 5–10 billion ZFN-modified CD4+ T cells. The clinical outcome was published in 2014^270^ and indicated that CCR5-knockout cells were protected from CCR5-tropic HIV infection, and the infusions of genetically engineered T cells into patients were well tolerated, with only 1 patient presenting with minor infusion-related adverse events. Since the preliminary demonstration of clinical safety, the main purpose of follow-up trials has been to further optimize the therapeutic effect of gene-edited T cells. Sangamo Therapeutics Inc. and the University of Pennsylvania tried to improve engraftment of the infused T cells by increasing the number of genetically modified CD4+ T cells, clearing nonmyeloablative lymphocytes, using multiple infusions of cells and switching from adenoviral vector delivery to mRNA electroporation. Although recent advances in ZFN-modified CD4+ T cell infusion have provided some evidence for the safety and low off-target rate of this therapy, a long-term evaluation is still needed. Another study provided proof for the safety of the permanent gene disruption of CCR5 in autologous CD34+ hematopoietic stem/progenitor cells (HSPCs) with ZFN ex vivo (NCT02500849). The main advantage of using HPSCs over T cells is that we will be able to obtain a large number of cell subsets that are protected from HIV infection, which are differentiated by the genetically edited CD34+ population. A recently reported article showed that Chinese scientists have established a CRISPR/Cas9-modified CCR5 gene editing system for adult HPSCs to achieve long-term and stable hematopoietic system reconstruction after infusion of modified CD34+ cells into patients with HIV-1 infection and ALL (NCT03164135).^271^ This study preliminarily proved the feasibility and safety of gene editing adult HPSC transplantation in the human body and would promote the development of gene editing technology in clinical applications. Because HSPC-based gene therapy is often confined by ex vivo culture techniques and difficulties in HPSC expansion, there is also interest in modifying patient-specific iPSCs and reprogramming them to HSPCs.^272^ (Clinical trials involving HPV and EBV infection are described in the Anticancer clinical trial section).

### Clinical trials of hematological diseases

To date, ZFN and CRISPR/Cas9 have been applied in five clinical gene-therapeutic trials pertaining to hematological diseases, including hemophilia B, β-thalassemia, and sickle cell disease.

Hemophilia B is a recessive, X-linked hemorrhagic disease represented by a lack of expression of coagulation factor IX (F IX).^273^ In November 2016, Sangamo Therapeutics Inc. initiated a phase I clinical trial (NCT02695160) with the expected 12 participants using SB-FIX, which is an AAV-delivered ZFN, designed to be intravenously delivered to the subject’s own hepatocytes to insert a corrective FIX transgene into the albumin locus; thus, they aim to achieve permanent FIX clotting factor production in the liver of severe hemophilia B patients. This ascending dose phase I study attempts to assess the safety and tolerability of SB-FIX in treating hemophilia B patients and is expected to be complete in January 2021. Abnormality in the β-globin gene (HBB) can reduce the synthesis of β-globin chains in hemoglobin, causing β-thalassemia.^274^ In January 2019, Allife Medical Science and Technology Co., Ltd. started a 12-subject early phase I trial, where they applied CRISPR/Cas9 to correct the HBB gene in vitro in patient-specific induced hematopoietic stem cells (iHSCs), and intravenously transfused the edited cells back to the HBB-mutated β-thalassemia subjects. This trial is expected to be complete in 2021. BCL11A, a key modifier in hemoglobin disorders characterized by repressing fetal hemoglobin (HbF), is associated with the clinical severity of β-thalassemia and sickle cell disease.^275^ Hence, gene therapy targeting BCL11A to treat the two diseases above has been tested in trials. Until now, three trials have tried to suppress the BCL11A gene in autologous CD34+ hematopoietic stem/progenitor cells in vitro and then intravenously transfuse the modified cells back to the subjects; all three trials initiated in 2018 and are expected to be complete in 2020–2022. Sangamo Therapeutics Inc. has led the first trial, NCT03432364, a single-dose phase I/II study with 6 subjects of transfusion-dependent β-thalassemia (TDT). ZFN has been applied to generate the gene-edited therapeutic cell ST-400; its safety, tolerability, and effects on HbF are to be evaluated and its transfusion requirements are to be assessed. Another single-dose phase I/II study trial (NCT03655678) with up to 45 subjects, focusing on transfusion-dependent β-thalassemia (TDT), was initiated by Vertex Pharmaceuticals Inc. They utilized CRISPR/Cas9-modified cell CTX001, aiming to test its safety and efficacy. With similar study designs and start and completion times, Vertex Pharmaceuticals Inc. have also tested CTX001 in severe sickle cell disease (NCT03745287).

### Clinical trials of hereditary eye diseases

Gene augmentation is successfully employed for the treatment of inherited retinal diseases, and a large number of clinical trials of gene augmentation are underway for LCA, choroideremia, achromatopsia, X-linked retinoschisis and RP.^276^ Until now, there has been only one clinical trial of gene editing in LCA10. Recently, a clinical study (NCT03872479) was initiated by Editas and Allergan to evaluate the safety, tolerability and efficacy of single-dose AGN-151587 (EDIT-101), an AAV vector containing 3 components: an *S. aureus* Cas9 and two gRNAs –gRNA-323 and gRNA-64. AGN-151587 could eliminate the mutation of c.2991 + 1655A > G in intron 26 of the CEP290 gene to treat LCA10. Although clinical trials on gene editing for ophthalmic diseases have just begun, the unique qualities of eyes, such as easy accessibility and relative immune-privileged status, make CRISPR–Cas a promising and available strategy for ophthalmic disease treatment in the near future.^164,276^

## Challenges in therapeutic targeting

In addition to the many benefits of genome editing, there are some technical challenges in translating these treatments to clinical disease therapy, primarily in terms of accuracy, efficacy and delivery hurdles. To cope with these challenges, scientists will need profound knowledge about the molecular nature of cancers, especially heterogeneous solid tumors, as well as carefully designed genome editing platforms in preclinical studies.

### Increasing the specificity of gene correction

The accuracy of gene editing technology is defined by the ability to edit the desired locus of interest within the genome. Mutations in undesired genomic loci, namely off-target effects, are inevitably rather pernicious, as they can lead to potential genomic toxicity, genome instability, the disruption of gene function, epigenetic alterations, and even carcinogenesis.^16,277,278^ Given that therapeutic gene targeting is strongly dependent on the creation of DSBs at specific target sites, assays of paramount importance have been developed to assess the targeting specificities of ZFNs, TALENs and Cas9 nucleases, such as in vitro selection libraries,^279,280^ mismatch-detection nuclease assay,^281^ newly reported high-throughput profiling,^282^ next-generation sequencing (NGS)^283^ and whole-genome sequencing (WGS).^284,285^ Thus, the above studies revealed a number of factors that might affect the specificity of gene editing, which can be roughly divided into two categories. First, the intrinsic specificity encoded in the Cas9 protein may determine the relative importance of each position that may differ between different sgRNA sequences. Second, the specificity also depends on the abundance of effective nuclease complexes relative to the target concentration.

Compared to ZFNs and TALENs, CRISPR/Cas9 may present higher potential for off-target effects in human cells.^278^ As previously noted, there is a tolerance of sequence mismatch when Cas9-sgRNA binds to the target DNA: both identical and highly homologous DNA sequences can be cleaved, leading to chromosomal rearrangements or off-target mutations.^286,287^ With numerous studies demonstrating the presence of its off-target activity, it has become the task with top priority to improve DNA specificity in CRISPR technology.^278^ Accordingly, several strategies have been exploited to minimize Cas9-mediated off-target effects and increase the cleavage specificity. Both the structure and composition of gRNA can affect the level of off‐target effects.^288,289^ A related method that has been reported to reduce the off-target effects induced by Cas9 is to choose unique target sequences that lack homology to other regions of the genome.^290^ In addition, the use of truncated and less-active sgRNAs that are shortened at the 5ʹ end by two to three nucleotides decreased undesired mutagenesis at some off-target sites because this sgRNA structure has higher sensitivity to mismatches.^277,282^ Another strategy to reduce the off-target effects is to harness a pair of nCas9 or RNA-guided FokI nucleases to generate paired nicks instead of DSBs, which can significantly avoid off-target cleavage without sacrificing genome editing efficiency.^291,292^ In addition, the concentration of Cas9-sgRNA delivered to cells should be carefully controlled, as it is another factor that affects off‐target effects.^293^ However, increasing specificity by reducing the amount of transfected DNA also results in reduced cleavage at the target. Therefore, a balance between on-target cleavage efficiency and off-target effects must be considered. Most recently, two different variants of monomeric *Streptococcus pyogenes* have been engineered to form a SpCas9 that exhibits improved genome-wide specificities. Slaymaker et al. described an enhanced SpCas9 that contains alanine substitutions at three positions and predicted the interaction of this variant with a nontarget DNA strand.^294^ In another study, Kleinstiver et al. created SpCas9-HF1 (high-fidelity variant 1) by introducing alanine substitutions at four residues in SpCas9 to disrupt nonspecific contacts with the phosphorylated framework of the target DNA strand, which interacts with gRNA.^295^ These engineered variants of SpCas9 have been engineered by reducing nonspecific interactions of proteins with different DNA strands, dramatically improving genome-wide specificity. They do not alter the target range or size of the DNA that is required to encode the desired Cas9 nuclease and a single gRNA; thus, functional mutations could also be combined to further increase specificity.

Alternative delivery methods have also been developed to improve the specificity of the editing process. Direct delivery of recombinant Cas9 protein and in vitro transcribed sgRNA either alone or in purified complexes reduced off-target effects when compared with plasmid transfected delivery systems.^296,297^ Anti-CRISPR molecules, recently discovered inhibitors for CRISPR systems, may add the precise control of genome editing strategy,^298^ which are currently tested.^299^

### Improving the efficiency of nuclease editing

The efficiency of DSB repair pathways mediated by NHEJ and HDR varies greatly between cell types and cell status; however, in most cases, NHEJ is more active than HDR. It has been observed that NHEJ is active throughout the cell cycle of a variety of cell types, including division and postmitosis.^11,300^ In contrast, HDR functions primarily in the S/G2 phase and is therefore largely restricted to actively dividing cells, limiting treatments for the precise genomic modification of mitotic cells.^301,302^ This difference makes the treatment of diseases that require genetic correction or gene insertion more challenging than those that require gene inactivation. Since NHEJ-mediated DSB repair can be applied to promote high levels of gene disruption in most cell types, the primary challenge to date has been to improve the efficiency of HDR.

Notably, recent studies have reported novel strategies to upregulate the efficiency of genome editing by inhibiting competing DNA repair pathways, primarily NHEJ-mediated DNA repair. Maruyama et al.^303^ successfully employed SCR7 to inhibit NHEJ by targeting a key enzyme (DNA ligase IV) in the NHEJ pathway, thereby increasing the genome editing efficiency in cell lines and mice by up to 19-fold. In another independent study, Kuhn et al. abolished NHEJ activity in human and mouse cell lines by the gene silencing of several key molecules of the NHEJ repair pathway (KU70, KU80 or DNA ligase IV), leading to increased genome editing efficiency.^304^ Further, Canny et al. discovered that 53BP1, a genetically encoded inhibitor, increased HDR-dependent genome editing efficiency by up to 5.6-fold through suppressing NHEJ activity in human and mouse cells.^305^ Interestingly, by application of an HDR enhancer, RS-1, Song et al. achieved multifold improvement on the CRISPR/Cas9- and TALEN-mediated knock-in efficiency both in vitro and in vivo, whereas the NHEJ inhibitor SCR7 has minimal effects.^306^ The identification of novel small molecule inhibitors against other NHEJ proteins, such as artemis and XRCC4, may further advance current strategies.^307,308^ An improved CRISPR system, called CRISPR/Cpf1 or CRISPR/Cas12a, that employs a smaller and simpler RNA-guided DNA nuclease, could target genomic regions that cannot be targeted by Cas9 and induce multiplex gene perturbation in vitro with frequencies of up to 45%.^309^ In addition, timed delivery of Cas9-guide RNP (RNA ribonucleoprotein) complexes was used to site-specifically induce DSBs and new genetic information, with high efficiency of HDR.^310^ In addition to the methods already mentioned, further research aimed at improving HDR efficiency will be necessary to optimize genome editing for a wider range of diseases.

Although the CRISPR/Cas9 gene editing system improves the efficiency of gene knockout and site-directed modification (including site-directed mutation and gene insertion), the efficiency of gene site-directed mutation based on a HR mechanism is still low. To improve the efficiency of site-directed mutation, the base editor (BE) system combining CRISPR/Cas9 and cytosine deaminase has been reported one after another.^311–313^ By using this system, the fusion protein composed of Cas9-cytidine deaminase and uracil glycosaminase inhibitor can be targeted at the desired site complementary to gRNA without double-stranded DNA fragmentation, and the amino group of pyrimidine (C) at the target site can be removed so that C becomes uracil (U), and U will be replaced by thymidine (T) with the replication of DNA. Finally, the single base C → T mutation is realized accurately and efficiently, leading to single-base-pair substitutions in eukaryotic cells.^314^ The BE technique adds an important tool to the research and application of genome editing technology.

### Optimizing the delivery system

**Fig. 4:**
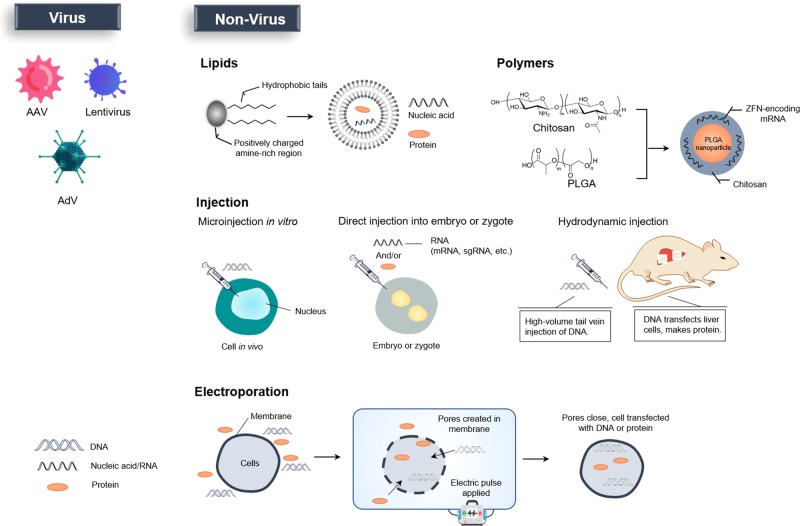
Viral and nonviral delivery systems for genome editing technology. The most commonly used viral vectors include adeno-associated viruses (AAVs), lentiviruses and adenoviruses (AdVs). Nonviral physical methods can be used for genome editing to deliver biomacromolecules intracellularly without the use of nanoparticles. Nonviral delivery may be microinjections in vitro, direct injection into the embryo or zygote ex vivo, or hydrodynamic injection in vivo. Alternatively, electroporation or mechanical deformation realize delivery by creating transient pores in the cellular membrane, making entry points for genome editing biomacromolecules.

One of the key challenges for the future application of gene editing tools will be the development of efficient and secure methods to deliver genetic editing elements, not only to the tumor cells ex vivo but also to somatic cells in vivo. Delivery methods include viral methods and nonviral physical methods (Fig. 4). Nonviral physical delivery methods, such as electroporation,^315^ hydrodynamic injection^316^ and lipid nanoparticles,^317^ have been widely utilized to deliver ZFNs, TALENs, and CRISPR in different cell lines and animal models. Despite their simplicity and safety, the relatively poor delivery efficacy limits the therapeutic applications of those nonviral delivery methods in vivo.^318^ In contrast, viral vectors (such as retroviruses, lentivirus, adenovirus (AdV) and AAV) have high delivery efficiency, and some of them have been approved for clinical uses.^319,320^ To date, viral delivery systems have been the most effective system for delivering plasmid-based nucleic acids to mammalian cells in vitro and in vivo, despite the possibility of introducing unintentional mutations and the existence of safety concerns.^321–324^ Recent studies have further highlighted other issues affecting delivery efficiency, including the immune risk of host tumors and cells to Cas9 proteins,^325^ as well as the DSB P53 responses related to genome editing.^326^ Many new viral and nonviral systems have been developed to overcome these problems.

Nonviral delivery systems could extend the range of genome editing therapies by alleviating concerns about the safety and immunogenicity of native cells in vivo. For instance, the delivery of plasmid DNA encoding a Cas9-sgRNA complex that targets VEGF using a PEG-PEI-cholesterol lipid polymer could achieve a gene knockout of approximately 50% in osteosarcoma cells in vitro and in vivo.^327^ A lipid delivery system containing PEG-poly lactic-coglycolic acid nanoparticles was used to deliver CRISPR DNA constructed by a CD68 promoter and achieve in vitro and in vivo gene editing of specific macrophages.^328^ Zuris and colleagues also studied lipid materials as vectors for genome-edited proteins. First, they fused Cas9 and TALEN into anionic GFP proteins to increase negative charges on the surface and then complexed them with Lipofectamine 2000TM (a commercially cationic lipofection reagent); this novel complex achieved 24% gene knockout of mouse embryonic stem cells in vitro and 13% gene knockout of mouse cochlea hair cells in vivo.^317^ The gene knockout rate of the complex to mouse embryonic stem cells in vitro was 24%.

According to Finn et al., lipid nanoparticles composed of PEG–lipids exhibited excellent serum stability. When used to deliver Cas9 mRNA and sgRNA targeting the mouse transthyretin gene in hepatocytes, they caused a drop in serum protein levels of more than 97%, which lasted for at least 12 months after a single systemic injection.^329^ Recent work by Cheng and Leong et al. has demonstrated that the delivery of Cas9 and sgRNA plasmids with cationic alpha-helical polypeptides is expected to enhance gene editing efficiency in vitro and in vivo. With this delivery system, repeated intratumoral injections in a HeLa xenograft mouse model resulted in ~67% targeted gene knockdown and >71% tumor growth inhibition and ultimately significantly prolonged the survival of tumor-bearing mice.^330^ Moreover, the Cas9 protein and sgRNA complex showed higher efficiency than plasmid-based CRISPR/Cas9 and Cas9 mRNA/sgRNA. For example, recombinant Cas9 proteins and sgRNA have been reported to achieve 16% editing efficiency in vitro through cell-penetrating peptide (CPP),^297,331^ while the delivery of purified Cas9 protein mediated by electroporation increased the editing efficiency to 79%,^296,332^ because transgenic proteins degraded rapidly and avoided long-lasting effects on the genome.

To improve the specificity and safety of viral-mediated gene editing delivery, different parts of preexisting viruses can be mixed together, creating hybrid virus vectors. The structure of the virus can be tweaked by point mutations, or the virus can incorporate small molecules, synthetic polymers and inorganic nanoparticles.^333^ For example, lentiviral vectors are typically pseudotyped with glycoprotein G from vesicular stomatitis virus (VSV-G), extending the vector tropism to a wide range of host cells.^334^ By controlling the ratio of assembled wild-type viral capsid to protease-activatable subunits, the overall transduction level of protease-activatable viruses (PAVs) increased.^335^ Using error-prone polymerase chain reaction (EP-PCR), Asuri et al. created a library of AAV capsid genes with point mutations, which resulted in a viral variant that was more efficient in delivering genetic payloads to human stem cells.^336^ The vector is further enhanced by conjugative delivery to ZFNs: the induced DSB facilitated HDR repair of the delivered transgene, thereby enabling gene targeting. Another way to further modify or enhance the functional properties of viruses is by incorporating synthetic nonbiological components such as polymers and nanoparticles. Hofherr et al.^337^ attached PEG-5000 to adenoviral vectors to generate adeno-PEG-injected counterparts (Ad-PEG). After intravenous injection into mammalian blood, PEGylation blunted the interactions of adenovirus with platelets and endothelial cells and reduced thrombocytopenia as well as D-dimer formation. In another study, Lee et al. investigated the possibility of conjugating the AAV surface-exposed lysine on the capsid with the activated PEG chains of PEG-2000 to protect the AAV vectors from neutralizing antibodies.^338^ At a critical conjugation ratio, the particles were moderately protected from serum neutralization by 2.3-fold over the unmodified vectors. These results indicate that certain modifications of viral vectors may have utility to reduce immune responses that are involved in the delivery process, thereby improving their safety for human gene therapy.

The proper selection of different delivery systems and CRISPR/Cas9 types also contributes to the reduction of off-target effects. For instance, the use of minicircle DNA is more efficient and less immunogenic than plasmid DNA per-mass due to the elimination of bacterial expression sequences.^339^ The codelivery of Cas9 and EGFR mutation-specific sgRNAs by adenovirus could precisely disrupt the oncogenic mutant allele, showing high specificity.^340^ Furthermore, nonviral polymers conjugated to the gold nanoparticle hybridization system have been recognized as a suitable vehicle for the delivery of Cas9 RNP complexes plus donor DNA, which could effectively correct the disease phenotypes of muscle cells after intramuscular injection.^341^

## Conclusions and future perspectives

The research evidence accumulated to date has demonstrated significant contributions of genome editing systems to exploit therapeutic strategies for various types of human diseases, among which the CRISPR/Cas9 system has been especially effective by directly interfering with target gene loci or deriving multifunctional tools. In the future, a combination of pooled CRISPR screening and the existing information on the genetic and epigenetic characteristics of cancer cell lines will be able to broadly identify synthetic lethal interactions in the genome and facilitate the discovery of novel drug targets. The CRISPR/Cas9 platform also provides a new tool to manipulate noncoding regions of the cancer genome, accelerating the functional exploration of aspects that are hitherto poorly characterized. The tremendous advances in the development of engineered nucleases (especially ZFNs, TALENs, and CRISPR/Cas9) paved the way for genome editing from a theoretical concept into clinical practice. At the end of 2017, Brian Madeux, an American man with Hunter’s syndrome, received a bold treatment at the Benioff Children’s Hospital at the University of California–San Francisco—the delivery of ZFNs via an AAV for in vivo genetic editing to treat his disease. This is the first report in the world describing the treatment of genetic diseases through in vivo gene editing, which further demonstrates that gene editing has extremely important clinical application potential for the treatment of genetic diseases. Genome editing technology has also been combined with tumor immunotherapy to provide more updated options for human disease treatment. As one of the most innovative and successful approaches in tumor immunotherapy, CAR T cell therapy was officially approved for use in the clinic in 2017. Refractory ALL and CLL patients responded completely to CAR T cell products directly targeting CD19, therefore the US Food and Drug Administration (FDA) recognized CAR T cell therapy as a “breakthrough therapy” and approved its treatment for leukemia and lymphoma. The effective response of CAR T therapy in clinical trials of B cell malignancies has evoked great enthusiasm for the ultimate intelligent treatment, brought hope to cancer patients, and led to the commercialization of CAR T cells by many pharmaceutical and biotechnology companies. However, the development of CAR T cell therapy is still in its infancy, and the high costs of CAR T cell therapy have made it unaffordable for a large population in society. Moreover, the commercial potential of this therapy, especially the possibility of becoming an off-the-shelf therapy, remains uncertain; in addition, its capacity to combat solid tumors remains to be confirmed.

At the same time, gene editing technology has also promoted the development of cell imaging, gene expression regulation, epigenetic modification, therapeutic drug development, functional gene screening, and gene diagnosis. Although the off-target effect in the implementation of gene editing technology still needs further optimization, innovative genome editing complexes and more specific nanostructured vehicles have improved efficiency and reduced toxicity during the delivery process, bringing genome editing technology closer to the clinic. With deeper exploration into this technology and the cooperation of the world scientific community, it is reasonable to believe that genome editing technology has the potential to ultimately elucidate biological mechanisms behind disease development and progression, thus providing novel therapies and finally promoting the development of the life sciences.

## References

[1] Rothstein RJ (1983). One-step gene disruption in yeast. Meth. Enzymol..

[2] Cornu, T. I., Mussolino, C. & Cathomen, T. Refining strategies to translate genome editing to the clinic. *Nat. Med.* **23**, 415–423 (2017). 10.1038/nm.4313 28388605

[3] Ghosh D, Venkataramani P, Nandi S, Bhattacharjee S (2019). CRISPR-Cas9 a boon or bane: the bumpy road ahead to cancer therapeutics. Cancer Cell Int..

[4] Gaj T, Gersbach CA, Barbas CF (2013). ZFN, TALEN, and CRISPR/Cas-based methods for genome engineering. Trends Biotechnol..

[5] Rouet P, Smih F, Jasin M (1994). Expression of a site-specific endonuclease stimulates homologous recombination in mammalian cells. Proc. Natl Acad. Sci. USA.

[6] Kosicki M, Tomberg K, Bradley A (2018). Repair of double-strand breaks induced by CRISPR-Cas9 leads to large deletions and complex rearrangements. Nat. Biotechnol..

[7] O’Driscoll M, Jeggo PA (2006). The role of double-strand break repair—insights from human genetics. Nat. Rev. Genet..

[8] Kaniecki Kyle, De Tullio Luisina, Greene Eric C. (2017). A change of view: homologous recombination at single-molecule resolution. Nature Reviews Genetics.

[9] Kim H, Kim JS (2014). A guide to genome engineering with programmable nucleases. Nat. Rev. Genet..

[10] Verma P, Greenberg RA (2016). Noncanonical views of homology-directed DNA repair. Genes Dev..

[11] Chang, H. H. Y., Pannunzio, N. R., Adachi, N. & Lieber, M. R. Non-homologous DNA end joining and alternative pathways to double-strand break repair. *Nat. Rev. Mol. Cell Biol*. **19**, 191–207 (2017). 10.1038/nrm.2017.48 PMC7062608 28512351

[12] Lieber MR, Gu J, Lu H, Shimazaki N, Tsai AG (2010). Nonhomologous DNA end joining (NHEJ) and chromosomal translocations in humans. Subcell. Biochem..

[13] Delacôte F, Lopez BS (2008). Importance of the cell cycle phase for the choice of the appropriate DSB repair pathway, for genome stability maintenance: the trans-S double-strand break repair model. Cell Cycle.

[14] Urnov FD, Rebar EJ, Holmes MC, Zhang HS, Gregory PD (2010). Genome editing with engineered zinc finger nucleases. Nat. Rev. Genet.

[15] Silva G (2011). Meganucleases and other tools for targeted genome engineering: perspectives and challenges for gene therapy. Curr. Gene Ther..

[16] Cathomen T, Keith Joung J (2008). Zinc-finger nucleases: the next generation emerges. Mol. Ther..

[17] Boch J (2009). Breaking the code of DNA binding specificity of TAL-type III effectors. Science.

[18] Zhang F (2011). Efficient construction of sequence-specific TAL effectors for modulating mammalian transcription. Nat. Biotechnol..

[19] Al-Attar S, Westra ER, van der Oost J, Brouns SJ (2011). Clustered regularly interspaced short palindromic repeats (CRISPRs): the hallmark of an ingenious antiviral defense mechanism in prokaryotes. Biol. Chem..

[20] Cox DB, Platt RJ, Zhang F (2015). Therapeutic genome editing: prospects and challenges. Nat. Med..

[21] Cong L (2013). Multiplex genome engineering using CRISPR/Cas systems. Science.

[22] Mali P (2013). RNA-guided human genome engineering via Cas9. Science.

[23] Kim YG, Cha J, Chandrasegaran S (1996). Hybrid restriction enzymes: zinc finger fusions to Fok I cleavage domain. Proc. Natl Acad. Sci. USA.

[24] Diakun GP, Fairall L, Klug A (1986). EXAFS study of the zinc-binding sites in the protein transcription factor IIIA. Nature.

[25] Beerli RR, Barbas CF (2002). Engineering polydactyl zinc-finger transcription factors. Nat. Biotechnol..

[26] Beerli RR, Schopfer U, Dreier B, Barbas CF (2000). Chemically regulated zinc finger transcription factors. J. Biol. Chem..

[27] Buck-Koehntop BA (2012). Molecular basis for recognition of methylated and specific DNA sequences by the zinc finger protein Kaiso. Proc. Natl Acad. Sci. USA.

[28] Fairall L, Schwabe JW, Chapman L, Finch JT, Rhodes D (1993). The crystal structure of a two zinc-finger peptide reveals an extension to the rules for zinc-finger/DNA recognition. Nature.

[29] Guo J, Gaj T, Barbas CF (2010). Directed evolution of an enhanced and highly efficient FokI cleavage domain for zinc finger nucleases. J. Mol. Biol..

[30] Smith J (2000). Requirements for double-strand cleavage by chimeric restriction enzymes with zinc finger DNA-recognition domains. Nucleic Acids Res..

[31] Beumer KJ (2008). Efficient gene targeting in *Drosophila* by direct embryo injection with zinc-finger nucleases. Proc. Natl Acad. Sci. USA.

[32] Paschon DE (2019). Diversifying the structure of zinc finger nucleases for high-precision genome editing. Nat. Commun..

[33] Bogdanove AJ, Schornack S, Lahaye T (2010). TAL effectors: finding plant genes for disease and defense. Curr. Opin. Plant Biol..

[34] Bogdanove AJ, Voytas DF (2011). TAL effectors: customizable proteins for DNA targeting. Science.

[35] Moscou MJ, Bogdanove AJ (2009). A simple cipher governs DNA recognition by TAL effectors. Science.

[36] Li T (2011). TAL nucleases (TALNs): hybrid proteins composed of TAL effectors and FokI DNA-cleavage domain. Nucleic Acids Res..

[37] Mussolino C (2011). A novel TALE nuclease scaffold enables high genome editing activity in combination with low toxicity. Nucleic Acids Res..

[38] Mercer AC, Gaj T, Fuller RP, Barbas CF (2012). Chimeric TALE recombinases with programmable DNA sequence specificity. Nucleic Acids Res..

[39] Cermak T (2011). Efficient design and assembly of custom TALEN and other TAL effector-based constructs for DNA targeting. Nucleic Acids Res..

[40] Reyon D (2012). FLASH assembly of TALENs for high-throughput genome editing. Nat. Biotechnol..

[41] Briggs AW (2012). Iterative capped assembly: rapid and scalable synthesis of repeat-module DNA such as TAL effectors from individual monomers. Nucleic Acids Res..

[42] Schmid-Burgk JL, Schmidt T, Kaiser V, Höning K, Hornung V (2013). A ligation-independent cloning technique for high-throughput assembly of transcription activator–like effector genes. Nat. Biotechnol..

[43] Ishino Y, Shinagawa H, Makino K, Amemura M, Nakata A (1987). Nucleotide sequence of the iap gene, responsible for alkaline phosphatase isozyme conversion in Escherichia coli, and identification of the gene product. J. Bacteriol..

[44] Jinek M (2012). A programmable dual-RNA-guided DNA endonuclease in adaptive bacterial immunity. Science.

[45] Bolotin A, Quinquis B, Sorokin A, Ehrlich SD (2005). Clustered regularly interspaced short palindrome repeats (CRISPRs) have spacers of extrachromosomal origin. Microbiology (Reading, England).

[46] Pourcel C, Salvignol G, Vergnaud G (2005). CRISPR elements in Yersinia pestis acquire new repeats by preferential uptake of bacteriophage DNA, and provide additional tools for evolutionary studies. Microbiology (Reading England).

[47] Makarova KS (2015). An updated evolutionary classification of CRISPR-Cas systems. Nat. Rev. Microbiol..

[48] Makarova KS (2011). Evolution and classification of the CRISPR-Cas systems. Nat. Rev. Microbiol..

[49] Jiang W, Bikard D, Cox D, Zhang F, Marraffini LA (2013). RNA-guided editing of bacterial genomes using CRISPR-Cas systems. Nat. Biotechnol..

[50] Sternberg SH, Redding S, Jinek M, Greene EC, Doudna JA (2014). DNA interrogation by the CRISPR RNA-guided endonuclease Cas9. Nature.

[51] Deveau H (2008). Phage response to CRISPR-encoded resistance in *Streptococcus thermophilus*. J. Bacteriol..

[52] Gasiunas G, Barrangou R, Horvath P, Siksnys V (2012). Cas9-crRNA ribonucleoprotein complex mediates specific DNA cleavage for adaptive immunity in bacteria. Proc. Natl Acad. Sci. USA.

[53] Ran FA (2013). Genome engineering using the CRISPR-Cas9 system. Nat. Protoc..

[54] Doudna JA, Charpentier E (2014). Genome editing. The new frontier of genome engineering with CRISPR-Cas9. Science.

[55] Liu, C., Zhang, L., Liu, H. & Cheng, K. Delivery strategies of the CRISPR-Cas9 gene-editing system for therapeutic applications. *J. Control. Release.* **266***,* 17–26 (2017). 10.1016/j.jconrel.2017.09.012 PMC5723556 28911805

[56] Biagioni A (2018). Delivery systems of CRISPR/Cas9-based cancer gene therapy. J. Biol. Eng..

[57] Abudayyeh Omar O., Gootenberg Jonathan S., Essletzbichler Patrick, Han Shuo, Joung Julia, Belanto Joseph J., Verdine Vanessa, Cox David B. T., Kellner Max J., Regev Aviv, Lander Eric S., Voytas Daniel F., Ting Alice Y., Zhang Feng (2017). RNA targeting with CRISPR–Cas13. Nature.

[58] Merkle Tobias, Merz Sarah, Reautschnig Philipp, Blaha Andreas, Li Qin, Vogel Paul, Wettengel Jacqueline, Li Jin Billy, Stafforst Thorsten (2019). Precise RNA editing by recruiting endogenous ADARs with antisense oligonucleotides. Nature Biotechnology.

[59] Tucker BA (2011). Transplantation of adult mouse iPS cell-derived photoreceptor precursors restores retinal structure and function in degenerative mice. PloS ONE.

[60] Homma K (2013). Developing rods transplanted into the degenerating retina of Crx-knockout mice exhibit neural activity similar to native photoreceptors. Stem Cells (Dayton, OH).

[61] Cai B (2018). Application of CRISPR/Cas9 technologies combined with iPSCs in the study and treatment of retinal degenerative diseases. Hum. Genet..

[62] Vogelstein B (2013). Cancer genome landscapes. Science.

[63] Choo Y, Sánchez-García I, Klug A (1994). In vivo repression by a site-specific DNA-binding protein designed against an oncogenic sequence. Nature.

[64] Do TU, Ho B, Shih SJ, Vaughan A (2012). Zinc finger nuclease induced DNA double stranded breaks and rearrangements in MLL. Mutat. Res..

[65] Provasi E (2012). Editing T cell specificity towards leukemia by zinc finger nucleases and lentiviral gene transfer. Nat. Med..

[66] Tanaka A (2013). A novel therapeutic molecule against HTLV-1 infection targeting provirus. Leukemia.

[67] Huang N (2018). Induction of apoptosis in imatinib sensitive and resistant chronic myeloid leukemia cells by efficient disruption of bcr-abl oncogene with zinc finger nucleases. J. Exp. Clin. Cancer Res..

[68] Piganeau M (2013). Cancer translocations in human cells induced by zinc finger and TALE nucleases. Genome Res..

[69] Puria R, Sahi S, Nain V (2012). HER2+ breast cancer therapy: by CPP-ZFN mediated targeting of mTOR?. Technol. Cancer Res. Treat..

[70] Herrmann F (2011). p53 Gene repair with zinc finger nucleases optimised by yeast 1-hybrid and validated by Solexa sequencing. PLoS ONE.

[71] Reik A, Zhou Yuanyue, Mendel MatthewC (2008). Zinc finger nucleases targeting the glucocorticoid receptor Allow IL-13 zetakine transgenic CTLs to kill glioblastoma cells in vivo in the presence of immunosuppressing glucocorticoids. Mol. Ther..

[72] Marchiq I, Le Floch R, Roux D, Simon MP, Pouyssegur J (2015). Genetic disruption of lactate/H+ symporters (MCTs) and their subunit CD147/BASIGIN sensitizes glycolytic tumor cells to phenformin. Cancer Res..

[73] Miller JC (2011). A TALE nuclease architecture for efficient genome editing. Nat. Biotechnol..

[74] Poirot Laurent, Philip Brian, Schiffer-Mannioui Cécile, Le Clerre Diane, Chion-Sotinel Isabelle, Derniame Sophie, Potrel Pierrick, Bas Cécile, Lemaire Laetitia, Galetto Roman, Lebuhotel Céline, Eyquem Justin, Cheung Gordon Weng-Kit, Duclert Aymeric, Gouble Agnès, Arnould Sylvain, Peggs Karl, Pule Martin, Scharenberg Andrew M., Smith Julianne (2015). Multiplex Genome-Edited T-cell Manufacturing Platform for “Off-the-Shelf” Adoptive T-cell Immunotherapies. Cancer Research.

[75] Wang J (2015). TALENs-mediated gene disruption of FLT3 in leukemia cells: Using genome-editing approach for exploring the molecular basis of gene abnormality. Sci. Rep..

[76] Nyquist MD (2013). TALEN-engineered AR gene rearrangements reveal endocrine uncoupling of androgen receptor in prostate cancer. Proc. Natl Acad. Sci. USA.

[77] Cai Y (2016). Loss of chromosome 8p governs tumor progression and drug response by altering lipid metabolism. Cancer Cell.

[78] Xiao L (2018). LRH-1 drives hepatocellular carcinoma partially through induction of c-myc and cyclin E1, and suppression of p21. Cancer Manag. Res..

[79] Zhan T, Rindtorff N, Betge J, Ebert MP, Boutros M (2019). CRISPR/Cas9 for cancer research and therapy. Semin Cancer Biol..

[80] Heckl Dirk, Kowalczyk Monika S, Yudovich David, Belizaire Roger, Puram Rishi V, McConkey Marie E, Thielke Anne, Aster Jon C, Regev Aviv, Ebert Benjamin L (2014). Generation of mouse models of myeloid malignancy with combinatorial genetic lesions using CRISPR-Cas9 genome editing. Nature Biotechnology.

[81] Sánchez-Rivera FJ, Jacks T (2015). Applications of the CRISPR-Cas9 system in cancer biology. Nat. Rev. Cancer.

[82] Sayin, V. I. & Papagiannakopoulos, T. Application of CRISPR-mediated genome engineering in cancer research. *Cancer Lett*. **387**, 10–17 (2016). 10.1016/j.canlet.2016.03.029 27000990

[83] Matano M (2015). Modeling colorectal cancer using CRISPR-Cas9-mediated engineering of human intestinal organoids. Nat. Med..

[84] Roper J (2018). Colonoscopy-based colorectal cancer modeling in mice with CRISPR-Cas9 genome editing and organoid transplantation. Nat. Protoc..

[85] Li AH (2015). Analysis of loss-of-function variants and 20 risk factor phenotypes in 8,554 individuals identifies loci influencing chronic disease. Nat. Genet..

[86] Abrahimi Parwiz, Chang William G., Kluger Martin S., Qyang Yibing, Tellides George, Saltzman W. Mark, Pober Jordan S. (2015). Efficient Gene Disruption in Cultured Primary Human Endothelial Cells by CRISPR/Cas9. Circulation Research.

[87] Carroll KJ (2016). A mouse model for adult cardiac-specific gene deletion with CRISPR/Cas9. Proc. Natl Acad. Sci. USA.

[88] Willer CJ (2008). Newly identified loci that influence lipid concentrations and risk of coronary artery disease. Nat. Genet..

[89] Gifford CA (2019). Oligogenic inheritance of a human heart disease involving a genetic modifier. Science.

[90] Yang D (2011). Generation of PPARγ mono-allelic knockout pigs via zinc-finger nucleases and nuclear transfer cloning. Cell Res..

[91] Umeyama K (2016). Generation of heterozygous fibrillin-1 mutant cloned pigs from genome-edited foetal fibroblasts. Sci. Rep..

[92] Ang YS (2016). Disease model of GATA4 mutation reveals transcription factor cooperativity in human cardiogenesis. Cell.

[93] Wang G (2014). Modeling the mitochondrial cardiomyopathy of Barth syndrome with induced pluripotent stem cell and heart-on-chip technologies. Nat. Med..

[94] Wallace Eimear, Howard Linda, Liu Min, O’Brien Timothy, Ward Deirdre, Shen Sanbing, Prendiville Terence (2019). Long QT Syndrome: Genetics and Future Perspective. Pediatric Cardiology.

[95] Burnett JR, Hooper AJ (2018). PCSK9—a journey to cardiovascular outcomes. N. Engl. J. Med..

[96] Pollin TI (2008). A null mutation in human APOC3 confers a favorable plasma lipid profile and apparent cardioprotection. Science.

[97] Ding Q (2014). Permanent alteration of PCSK9 with in vivo CRISPR-Cas9 genome editing. Circ. Res..

[98] Crosby J (2014). Loss-of-function mutations in APOC3, triglycerides, and coronary disease. N. Engl. J. Med..

[99] Zhan Yongkun, Sun Xiaolei, Li Bin, Cai Huanhuan, Xu Chen, Liang Qianqian, Lu Chao, Qian Ruizhe, Chen Sifeng, Yin Lianhua, Sheng Wei, Huang Guoying, Sun Aijun, Ge Junbo, Sun Ning (2018). Establishment of a PRKAG2 cardiac syndrome disease model and mechanism study using human induced pluripotent stem cells. Journal of Molecular and Cellular Cardiology.

[100] O’Rahilly S (2009). Human genetics illuminates the paths to metabolic disease. Nature.

[101] Coppari R, Bjørbæk C (2012). Leptin revisited: its mechanism of action and potential for treating diabetes. Nat. Rev. Drug Disco..

[102] Giesbertz P (2015). Metabolite profiling in plasma and tissues of ob/ob and db/db mice identifies novel markers of obesity and type 2 diabetes. Diabetologia.

[103] Chen Yuting, Lu Wenqing, Gao Na, Long Yi, Shao Yanjiao, Liu Meizhen, Chen Huaqing, Ye Shixin, Ma Xueyun, Liu Mingyao, Li Dali (2016). Generation of obese rat model by transcription activator-like effector nucleases targeting the leptin receptor gene. Science China Life Sciences.

[104] Bao D (2015). Preliminary characterization of a leptin receptor knockout rat created by CRISPR/Cas9 system. Sci. Rep..

[105] Wang X (2016). Characterization of novel cytochrome P450 2E1 knockout rat model generated by CRISPR/Cas9. Biochem. Pharmacol..

[106] Claussnitzer Melina, Dankel Simon N., Kim Kyoung-Han, Quon Gerald, Meuleman Wouter, Haugen Christine, Glunk Viktoria, Sousa Isabel S., Beaudry Jacqueline L., Puviindran Vijitha, Abdennur Nezar A., Liu Jannel, Svensson Per-Arne, Hsu Yi-Hsiang, Drucker Daniel J., Mellgren Gunnar, Hui Chi-Chung, Hauner Hans, Kellis Manolis (2015). FTO Obesity Variant Circuitry and Adipocyte Browning in Humans. New England Journal of Medicine.

[107] Naylor, J. et al. Use of CRISPR/Cas-9 engineered INS-1 pancreatic beta cells to define the pharmacology of dual GIPR/GLP-1R agonists. *Biochem. J*. **473**, 2881–2891 (2016). 10.1042/BCJ20160476 27422784

[108] Vethe H (2017). Probing the missing mature β-cell proteomic landscape in differentiating patient iPSC-derived cells. Sci. Rep..

[109] Liao, H. K. et al. In Vivo Target Gene Activation via CRISPR/Cas9-Mediated Trans-epigenetic Modulation. *Cell* **171**, 1495–1507.e15 (2017). 10.1016/j.cell.2017.10.025 PMC5732045 29224783

[110] Tirronen, A., Hokkanen, K., Vuorio, T. & Ylä-Herttuala, S. Recent advances in novel therapies for lipid disorders. *Hum. Mol. Genet*. **28**, R49–R54 (2019). 10.1093/hmg/ddz132 31227825

[111] Nakagawa Y (2016). Hyperlipidemia and hepatitis in liver-specific CREB3L3 knockout mice generated using a one-step CRISPR/Cas9 system. Sci. Rep..

[112] Carlson DF (2012). Efficient TALEN-mediated gene knockout in livestock. Proc. Natl Acad. Sci. USA.

[113] Burkhardt R (2010). Trib1 is a lipid- and myocardial infarction-associated gene that regulates hepatic lipogenesis and VLDL production in mice. J. Clin. Invest..

[114] Nagiec MM (2015). Modulators of hepatic lipoprotein metabolism identified in a search for small-molecule inducers of tribbles pseudokinase 1 expression. PLoS ONE.

[115] Nance MA (2017). Genetics of Huntington disease. Handb. Clin. Neurol..

[116] Wood LB, Winslow AR, Strasser SD (2015). Systems biology of neurodegenerative diseases. Integr. Biol. (Camb.).

[117] Soto, C. & Pritzkow, S. Protein misfolding, aggregation, and conformational strains in neurodegenerative diseases. *Nat. Neurosci*. **21**, 1332–1340 (2018). 10.1038/s41593-018-0235-9 PMC6432913 30250260

[118] Hu Z, Yang B, Mo X, Xiao H (2015). Mechanism and regulation of autophagy and its role in neuronal diseases. Mol. Neurobiol..

[119] Sahebkar, A., Panahi, Y., Yaribeygi, H. & Javadi, B. Oxidative stress in neurodegenerative diseases: a review. *Mol Neurobiol.* **53***,* 4094–4125 (2018). 10.1007/s12035-015-9337-5 PMC4937091 26198567

[120] Rossi F, Cattaneo E (2002). Opinion: neural stem cell therapy for neurological diseases: dreams and reality. Nat. Rev. Neurosci..

[121] Fan HC (2018). The role of gene editing in neurodegenerative diseases. Cell Transpl..

[122] Garriga-Canut M (2012). Synthetic zinc finger repressors reduce mutant huntingtin expression in the brain of R6/2 mice. Proc. Natl Acad. Sci. USA.

[123] An MC (2012). Genetic correction of Huntington’s disease phenotypes in induced pluripotent stem cells. Cell Stem Cell.

[124] Xu Xiaohong, Tay Yilin, Sim Bernice, Yoon Su-In, Huang Yihui, Ooi Jolene, Utami Kagistia Hana, Ziaei Amin, Ng Bryan, Radulescu Carola, Low Donovan, Ng Alvin Yu Jin, Loh Marie, Venkatesh Byrappa, Ginhoux Florent, Augustine George J., Pouladi Mahmoud A. (2017). Reversal of Phenotypic Abnormalities by CRISPR/Cas9-Mediated Gene Correction in Huntington Disease Patient-Derived Induced Pluripotent Stem Cells. Stem Cell Reports.

[125] Yan Sen, Tu Zhuchi, Liu Zhaoming, Fan Nana, Yang Huiming, Yang Su, Yang Weili, Zhao Yu, Ouyang Zhen, Lai Chengdan, Yang Huaqiang, Li Li, Liu Qishuai, Shi Hui, Xu Guangqing, Zhao Heng, Wei Hongjiang, Pei Zhong, Li Shihua, Lai Liangxue, Li Xiao-Jiang (2018). A Huntingtin Knockin Pig Model Recapitulates Features of Selective Neurodegeneration in Huntington’s Disease. Cell.

[126] Di Fede G (2009). A recessive mutation in the APP gene with dominant-negative effect on amyloidogenesis. Science.

[127] Jonsson T (2012). A mutation in APP protects against Alzheimer’s disease and age-related cognitive decline. Nature.

[128] Giaccone G (2010). Neuropathology of the recessive A673V APP mutation: Alzheimer disease with distinctive features. Acta Neuropathol..

[129] Martiskainen Henna, Herukka Sanna-Kaisa, Stančáková Alena, Paananen Jussi, Soininen Hilkka, Kuusisto Johanna, Laakso Markku, Hiltunen Mikko (2017). Decreased plasma β-amyloid in the Alzheimer's disease APP A673T variant carriers. Annals of Neurology.

[130] Wang Y (2016). Lost region in amyloid precursor protein (APP) through TALEN-mediated genome editing alters mitochondrial morphology. Sci. Rep..

[131] Paquet D (2016). Efficient introduction of specific homozygous and heterozygous mutations using CRISPR/Cas9. Nature.

[132] Abudayyeh Omar O., Gootenberg Jonathan S., Franklin Brian, Koob Jeremy, Kellner Max J., Ladha Alim, Joung Julia, Kirchgatterer Paul, Cox David B. T., Zhang Feng (2019). A cytosine deaminase for programmable single-base RNA editing. Science.

[133] Lubbe S, Morris HR (2014). Recent advances in Parkinson’s disease genetics. J. Neurol..

[134] Dansithong W, Paul S, Scoles DR, Pulst SM, Huynh DP (2015). Generation of SNCA cell models using zinc finger nuclease (ZFN) technology for efficient high-throughput drug screening. PLoS ONE.

[135] Reinhardt P (2013). Genetic correction of a LRRK2 mutation in human iPSCs links parkinsonian neurodegeneration to ERK-dependent changes in gene expression. Cell Stem Cell.

[136] Soldner F (2016). Parkinson-associated risk variant in distal enhancer of α-synuclein modulates target gene expression. Nature.

[137] Chen, S., Yu, X. & Guo, D. CRISPR-Cas targeting of host genes as an antiviral strategy. *Viruses* **10**, 40 (2018). 10.3390/v10010040 PMC5795453 29337866

[138] Perez EE (2008). Establishment of HIV-1 resistance in CD4+ T cells by genome editing using zinc-finger nucleases. Nat. Biotechnol..

[139] Hütter G (2009). Long-term control of HIV by CCR5 Delta32/Delta32 stem-cell transplantation. N. Engl. J. Med..

[140] Allers K (2011). Evidence for the cure of HIV infection by CCR5Δ32/Δ32 stem cell transplantation. Blood.

[141] Allen AG (2018). Gene editing of HIV-1 Co-receptors to prevent and/or cure virus infection. Front Microbiol.

[142] Didigu CA (2014). Simultaneous zinc-finger nuclease editing of the HIV coreceptors ccr5 and cxcr4 protects CD4+ T cells from HIV-1 infection. Blood.

[143] Mussolino C (2014). TALENs facilitate targeted genome editing in human cells with high specificity and low cytotoxicity. Nucleic Acids Res..

[144] Ebina H, Misawa N, Kanemura Y, Koyanagi Y (2013). Harnessing the CRISPR/Cas9 system to disrupt latent HIV-1 provirus. Sci. Rep..

[145] Mandal PK (2014). Efficient ablation of genes in human hematopoietic stem and effector cells using CRISPR/Cas9. Cell Stem Cell.

[146] Hendel Ayal, Bak Rasmus O, Clark Joseph T, Kennedy Andrew B, Ryan Daniel E, Roy Subhadeep, Steinfeld Israel, Lunstad Benjamin D, Kaiser Robert J, Wilkens Alec B, Bacchetta Rosa, Tsalenko Anya, Dellinger Douglas, Bruhn Laurakay, Porteus Matthew H (2015). Chemically modified guide RNAs enhance CRISPR-Cas genome editing in human primary cells. Nature Biotechnology.

[147] Hu W (2014). RNA-directed gene editing specifically eradicates latent and prevents new HIV-1 infection. Proc. Natl Acad. Sci. USA.

[148] Moody CA, Laimins LA (2010). Human papillomavirus oncoproteins: pathways to transformation. Nat. Rev. Cancer.

[149] Ding W (2014). Zinc finger nucleases targeting the human papillomavirus E7 oncogene induce E7 disruption and a transformed phenotype in HPV16/18-positive cervical cancer cells. Clin. Cancer Res..

[150] Ren Ci, Gao Chun, Li Xiaomin, Shen Hui, Wang Liming, Zhu Da, Wu Peng, Ma Ding, Wang Hui, Ding Wencheng (2018). Zinc Finger Nuclease Combines with Cisplatin and Trichostatin A Enhances the Antitumor Potency in Cervical Cancer Cells. Anti-Cancer Agents in Medicinal Chemistry.

[151] Wayengera M (2012). Zinc finger arrays binding human papillomavirus types 16 and 18 genomic DNA: precursors of gene-therapeutics for in-situ reversal of associated cervical neoplasia. Theor. Biol. Med. Model.

[152] Hu Z (2015). TALEN-mediated targeting of HPV oncogenes ameliorates HPV-related cervical malignancy. J. Clin. Invest.

[153] Zhen S (2016). In vitro and in vivo synergistic therapeutic effect of cisplatin with human papillomavirus16 E6/E7 CRISPR/Cas9 on cervical cancer cell line. Transl. Oncol..

[154] Cradick TJ, Keck K, Bradshaw S, Jamieson AC, McCaffrey AP (2010). Zinc-finger nucleases as a novel therapeutic strategy for targeting hepatitis B virus DNAs. Mol. Ther..

[155] Ramanan V (2015). CRISPR/Cas9 cleavage of viral DNA efficiently suppresses hepatitis B virus. Sci. Rep..

[156] Wang J, Quake SR (2014). RNA-guided endonuclease provides a therapeutic strategy to cure latent herpesviridae infection. Proc. Natl Acad. Sci. USA.

[157] Cai, S. W., Zhang, Y., Hou, M. Z., Liu, Y. & Li, X. R. The research advances and applications of genome editing in hereditary eye diseases. *Zhonghua Yan Ke Za Zhi* **53**, 386–391 (2017). 10.3760/cma.j.issn.0412-4081.2017.05.014 28494568

[158] Bjork S, Hurt CM, Ho VK, Angelotti T (2013). REEPs are membrane shaping adapter proteins that modulate specific g protein-coupled receptor trafficking by affecting ER cargo capacity. PloS ONE.

[159] Arno G (2016). Mutations in REEP6 cause autosomal-recessive retinitis pigmentosa. Am. J. Hum. Genet..

[160] Bowes C (1990). Retinal degeneration in the rd mouse is caused by a defect in the beta subunit of rod cGMP-phosphodiesterase. Nature.

[161] Keeler CE (1928). THE geotropic reaction of rodless mice in light and in darkness. J. Gen. Physiol..

[162] Wu WH (2016). CRISPR repair reveals causative mutation in a preclinical model of retinitis pigmentosa. Mol. Ther.: J. Am. Soc. Gene Ther..

[163] McGill TJ (2012). Optomotor and immunohistochemical changes in the juvenile S334ter rat. Exp. Eye Res..

[164] Cho GY (2017). CRISPR-mediated ophthalmic genome surgery. Curr. Ophthalmol. Rep..

[165] Bakondi B (2016). In Vivo CRISPR/Cas9 gene editing corrects retinal dystrophy in the S334ter-3 rat model of autosomal dominant retinitis pigmentosa. Mol. Ther.: J. Am. Soc. Gene Ther..

[166] Latella MC (2016). In vivo editing of the human mutant rhodopsin gene by electroporation of plasmid-based CRISPR/Cas9 in the mouse retina. Mol. Ther. Nucleic Acids.

[167] Suzuki K (2016). In vivo genome editing via CRISPR/Cas9 mediated homology-independent targeted integration. Nature.

[168] Bassuk AG, Zheng A, Li Y, Tsang SH, Mahajan VB (2016). Precision medicine: genetic repair of retinitis pigmentosa in patient-derived stem cells. Sci. Rep..

[169] Deng WL (2018). Gene correction reverses ciliopathy and photoreceptor loss in iPSC-derived retinal organoids from retinitis pigmentosa patients. Stem Cell Rep..

[170] Liao C, Zhang D, Mungo C, Tompkins DA, Zeidan AM (2014). Is diabetes mellitus associated with increased incidence and disease-specific mortality in endometrial cancer? A systematic review and meta-analysis of cohort studies. Gynecologic Oncol..

[171] Zhong H, Chen Y, Li Y, Chen R, Mardon G (2015). CRISPR-engineered mosaicism rapidly reveals that loss of Kcnj13 function in mice mimics human disease phenotypes. Sci. Rep..

[172] Ruan GX (2017). CRISPR/Cas9-mediated genome editing as a therapeutic approach for leber congenital amaurosis 10. Mol. Ther.: J. Am. Soc. Gene Ther..

[173] Maeder ML (2019). Development of a gene-editing approach to restore vision loss in Leber congenital amaurosis type 10. Nat. Med..

[174] Lee WH, Murphree AL, Benedict WF (1984). Expression and amplification of the N-myc gene in primary retinoblastoma. Nature.

[175] Solin SL, Shive HR, Woolard KD, Essner JJ, McGrail M (2015). Rapid tumor induction in zebrafish by TALEN-mediated somatic inactivation of the retinoblastoma1 tumor suppressor rb1. Sci. Rep..

[176] Naert T (2016). CRISPR/Cas9 mediated knockout of rb1 and rbl1 leads to rapid and penetrant retinoblastoma development in Xenopus tropicalis. Sci. Rep..

[177] Tu J (2018). Generation of human embryonic stem cell line with heterozygous RB1 deletion by CRIPSR/Cas9 nickase. Stem Cell Res.

[178] Hollands H (2013). Do findings on routine examination identify patients at risk for primary open-angle glaucoma? The rational clinical examination systematic review. Jama.

[179] Alward WL (1998). Clinical features associated with mutations in the chromosome 1 open-angle glaucoma gene (GLC1A). New Engl. J. Med..

[180] Stone EM (1997). Identification of a gene that causes primary open angle glaucoma. Science.

[181] Kim BS (2001). Targeted disruption of the myocilin gene (Myoc) suggests that human glaucoma-causing mutations are gain of function. Mol. Cell. Biol..

[182] Carbone MA (2009). Overexpression of myocilin in the *Drosophila* eye activates the unfolded protein response: implications for glaucoma. PloS ONE.

[183] Joe MK (2003). Accumulation of mutant myocilins in ER leads to ER stress and potential cytotoxicity in human trabecular meshwork cells. Biochem. Biophys. Res. Commun..

[184] Liu Y, Vollrath D (2004). Reversal of mutant myocilin non-secretion and cell killing: implications for glaucoma. Hum. Mol. Genet..

[185] Yam GH, Gaplovska-Kysela K, Zuber C, Roth J (2007). Aggregated myocilin induces russell bodies and causes apoptosis: implications for the pathogenesis of myocilin-caused primary open-angle glaucoma. Am. J. Pathol..

[186] Jain A (2017). CRISPR-Cas9-based treatment of myocilin-associated glaucoma. Proc. Natl Acad. Sci. USA.

[187] Liao H (2011). Development of allele-specific therapeutic siRNA in Meesmann epithelial corneal dystrophy. PloS ONE.

[188] Courtney DG (2016). CRISPR/Cas9 DNA cleavage at SNP-derived PAM enables both in vitro and in vivo KRT12 mutation-specific targeting. Gene Ther..

[189] Shima M (2016). Factor VIII-mimetic function of humanized bispecific antibody in hemophilia A. N. Engl. J. Med..

[190] Park CY (2015). Functional correction of large factor VIII gene chromosomal inversions in hemophilia a patient-derived iPSCs using CRISPR-Cas9. Cell Stem Cell.

[191] Guan Yuting, Ma Yanlin, Li Qi, Sun Zhenliang, Ma Lie, Wu Lijuan, Wang Liren, Zeng Li, Shao Yanjiao, Chen Yuting, Ma Ning, Lu Wenqing, Hu Kewen, Han Honghui, Yu Yanhong, Huang Yuanhua, Liu Mingyao, Li Dali (2016). CRISPR /Cas9‐mediated somatic correction of a novel coagulator factor IX gene mutation ameliorates hemophilia in mouse. EMBO Molecular Medicine.

[192] Hai T, Teng F, Guo R, Li W, Zhou Q (2014). One-step generation of knockout pigs by zygote injection of CRISPR/Cas system. Cell Res..

[193] Rees DC, Williams TN, Gladwin MT (2010). Sickle-cell disease. Lancet.

[194] Dever, D. P. et al. CRISPR/Cas9 β-globin gene targeting in human haematopoietic stem cells. *Nature* **539***,* 384–389 (2016). 10.1038/nature20134 PMC5898607 27820943

[195] Martin RM (2019). Highly efficient and marker-free genome editing of human pluripotent stem cells by CRISPR-Cas9 RNP and AAV6 donor-mediated homologous recombination. Cell Stem Cell.

[196] Verhaart, I. E. C. & Aartsma-Rus, A. Therapeutic developments for Duchenne muscular dystrophy. *Nat. Rev. Neurol.* **15**, 373–386 (2019). 10.1038/s41582-019-0203-3 31147635

[197] Larcher T (2014). Characterization of dystrophin deficient rats: a new model for Duchenne muscular dystrophy. PLoS ONE.

[198] Sui Tingting, Lau Yeh Siang, Liu Di, Liu Tingjun, Xu Li, Gao Yandi, Lai Liangxue, Li Zhanjun, Han Renzhi (2018). A novel rabbit model of Duchenne muscular dystrophy generated by CRISPR/Cas9. Disease Models & Mechanisms.

[199] Chen Y (2015). Generation of cynomolgus monkey chimeric fetuses using embryonic stem cells. Cell Stem Cell.

[200] Chen Y (2015). Functional disruption of the dystrophin gene in rhesus monkey using CRISPR/Cas9. Hum. Mol. Genet..

[201] Xu Li, Park Ki Ho, Zhao Lixia, Xu Jing, El Refaey Mona, Gao Yandi, Zhu Hua, Ma Jianjie, Han Renzhi (2016). CRISPR-mediated Genome Editing Restores Dystrophin Expression and Function in mdx Mice. Molecular Therapy.

[202] Bengtsson NE (2017). Muscle-specific CRISPR/Cas9 dystrophin gene editing ameliorates pathophysiology in a mouse model for Duchenne muscular dystrophy. Nat. Commun..

[203] Nelson, C. E. et al. In vivo genome editing improves muscle function in a mouse model of Duchenne muscular dystrophy. *Science* **351**, 403–407 (2016). 10.1126/science.aad5143 PMC4883596 26721684

[204] Pai SY (2014). Transplantation outcomes for severe combined immunodeficiency, 2000-2009. N. Engl. J. Med..

[205] Lombardo A (2007). Gene editing in human stem cells using zinc finger nucleases and integrase-defective lentiviral vector delivery. Nat. Biotechnol..

[206] Flisikowska T (2011). Efficient immunoglobulin gene disruption and targeted replacement in rabbit using zinc finger nucleases. PLoS ONE.

[207] Wang Y (2014). Generation of knockout rabbits using transcription activator-like effector nucleases. Cell Regen. (Lond.).

[208] Yan Q (2014). Generation of multi-gene knockout rabbits using the Cas9/gRNA system. Cell Regen. (Lond.).

[209] Zhou J (2014). One-step generation of different immunodeficient mice with multiple gene modifications by CRISPR/Cas9 mediated genome engineering. Int. J. Biochem. Cell Biol..

[210] Ott de Bruin LM, Volpi S, Musunuru K (2015). Novel genome-editing tools to model and correct primary immunodeficiencies. Front Immunol..

[211] Chen Y (2017). Modeling Rett syndrome using TALEN-Edited MECP2 mutant cynomolgus monkeys. Cell.

[212] Chen, J. R. et al. Effects of genetic correction on the differentiation of hair cell-like cells from iPSCs with MYO15A mutation. *Cell Death Differ*. **9**, eaaj2013 (2016). 10.1038/cdd.2016.16 PMC4947666 26915297

[213] Jiang W (2018). Production of Wilson disease model rabbits with homology-directed precision point mutations in the ATP7B gene using the CRISPR/Cas9 system. Sci. Rep..

[214] Kurome M (2014). 361 growth hormone receptor mutant pigs produced by using the clustered regularly interspaced short palindromic repeats (crispr) and crispr-associated systems in in vitro-produced zygotes. Reprod. Fertil. Dev..

[215] Tseng Wei-Chia, Loeb Hannah E., Pei Wuhong, Tsai-Morris Chon-Hwa, Xu Lisha, Cluzeau Celine V., Wassif Christopher A., Feldman Benjamin, Burgess Shawn M., Pavan William J., Porter Forbes D. (2018). Modeling Niemann-Pick disease type C1 in zebrafish: a robust platform forin vivoscreening of candidate therapeutic compounds. Disease Models & Mechanisms.

[216] Abdul-Wahab A, Qasim W, McGrath JA (2014). Gene therapies for inherited skin disorders. Semin Cutan. Med. Surg..

[217] Kirkwood JM (2012). Immunotherapy of cancer in 2012. CA Cancer J. Clin..

[218] Rein LAM, Yang H, Chao NJ (2018). Applications of gene editing technologies to cellular therapies. Biol. Blood Marrow Transpl..

[219] June CH, Sadelain M (2018). Chimeric antigen receptor therapy. N. Engl. J. Med.

[220] Sadelain M, Brentjens R, Rivière I (2009). The promise and potential pitfalls of chimeric antigen receptors. Curr. Opin. Immunol..

[221] Davenport AJ (2015). CAR-T cells inflict sequential killing of multiple tumor target cells. Cancer Immunol. Res.

[222] Zhao J, Lin Q, Song Y, Liu D (2018). Universal CARs, universal T cells, and universal CAR T cells. J. Hematol. Oncol..

[223] Torikai H (2012). A foundation for universal T-cell based immunotherapy: T cells engineered to express a CD19-specific chimeric-antigen-receptor and eliminate expression of endogenous TCR. Blood.

[224] Torikai H (2013). Toward eliminating HLA class I expression to generate universal cells from allogeneic donors. Blood.

[225] Qasim Waseem, Zhan Hong, Samarasinghe Sujith, Adams Stuart, Amrolia Persis, Stafford Sian, Butler Katie, Rivat Christine, Wright Gary, Somana Kathy, Ghorashian Sara, Pinner Danielle, Ahsan Gul, Gilmour Kimberly, Lucchini Giovanna, Inglott Sarah, Mifsud William, Chiesa Robert, Peggs Karl S., Chan Lucas, Farzaneh Farzin, Thrasher Adrian J., Vora Ajay, Pule Martin, Veys Paul (2017). Molecular remission of infant B-ALL after infusion of universal TALEN gene-edited CAR T cells. Science Translational Medicine.

[226] Wei G, Wang J, Huang H, Zhao Y (2017). Novel immunotherapies for adult patients with B-lineage acute lymphoblastic leukemia. J. Hematol. Oncol..

[227] Liu Xiaojuan, Zhang Yongping, Cheng Chen, Cheng Albert W, Zhang Xingying, Li Na, Xia Changqing, Wei Xiaofei, Liu Xiang, Wang Haoyi (2016). CRISPR-Cas9-mediated multiplex gene editing in CAR-T cells. Cell Research.

[228] Ren, J. et al. A versatile system for rapid multiplex genome-edited CAR T cell generation. *Oncotarget* **8**, 17002–17011 (2017). 10.18632/oncotarget.15218 PMC5370017 28199983

[229] Kenderian SS (2016). Identification of PD1 and TIM3 as checkpoints that limit chimeric antigen receptor T cell efficacy in leukemia. Biol. Blood Marrow Transplant..

[230] Leone RD, Emens LA (2018). Targeting adenosine for cancer immunotherapy. J. Immunother. Cancer.

[231] Zhang Yongping, Zhang Xingying, Cheng Chen, Mu Wei, Liu Xiaojuan, Li Na, Wei Xiaofei, Liu Xiang, Xia Changqing, Wang Haoyi (2017). CRISPR-Cas9 mediated LAG-3 disruption in CAR-T cells. Frontiers of Medicine.

[232] Wong Alan S. L., Choi Gigi C. G., Cui Cheryl H., Pregernig Gabriela, Milani Pamela, Adam Miriam, Perli Samuel D., Kazer Samuel W., Gaillard Aleth, Hermann Mario, Shalek Alex K., Fraenkel Ernest, Lu Timothy K. (2016). Multiplexed barcoded CRISPR-Cas9 screening enabled by CombiGEM. Proceedings of the National Academy of Sciences.

[233] Yu, J. S. L. & Yusa, K. Genome-wide CRISPR-Cas9 screening in mammalian cells. *Methods.* **164–165***,* 29–35 (2019). 10.1016/j.ymeth.2019.04.015 31034882

[234] Luo J (2016). CRISPR/Cas9: from genome engineering to cancer drug discovery. *Trends*. Cancer.

[235] Bester AC (2018). An integrated genome-wide CRISPRa approach to functionalize lncRNAs in drug resistance. Cell.

[236] Tzelepis K (2016). A CRISPR dropout screen identifies genetic vulnerabilities and therapeutic targets in acute myeloid leukemia. Cell Rep..

[237] Munoz, D. M. et al. CRISPR screens provide a comprehensive assessment of cancer vulnerabilities but generate false-positive hits for highly amplified genomic regions. *Cancer Discov* **6***.* 900–13. (2016). 10.1158/2159-8290.CD-16-0178 27260157

[238] Hart T (2015). High-resolution CRISPR screens reveal fitness genes and genotype-specific cancer liabilities. Cell.

[239] Wang Tim, Yu Haiyan, Hughes Nicholas W., Liu Bingxu, Kendirli Arek, Klein Klara, Chen Walter W., Lander Eric S., Sabatini David M. (2017). Gene Essentiality Profiling Reveals Gene Networks and Synthetic Lethal Interactions with Oncogenic Ras. Cell.

[240] Chen S (2015). Genome-wide CRISPR screen in a mouse model of tumor growth and metastasis. Cell.

[241] McFadden DG (2014). Genetic and clonal dissection of murine small cell lung carcinoma progression by genome sequencing. Cell.

[242] Tang JT (2011). MicroRNA 345, a methylation-sensitive microRNA is involved in cell proliferation and invasion in human colorectal cancer. Carcinogenesis.

[243] Cheng Z, Ma R, Tan W, Zhang L (2014). MiR-152 suppresses the proliferation and invasion of NSCLC cells by inhibiting FGF2. Exp. Mol. Med..

[244] Song, C. Q. et al. Genome-wide CRISPR Screen Identifies Regulators of MAPK as Suppressors of Liver Tumors in Mice. *Gastroenterology* **5***,* 1161–1173.e1 (2016). 10.1053/j.gastro.2016.12.002 PMC6204228 27956228

[245] Han Kyuho, Jeng Edwin E, Hess Gaelen T, Morgens David W, Li Amy, Bassik Michael C (2017). Synergistic drug combinations for cancer identified in a CRISPR screen for pairwise genetic interactions. Nature Biotechnology.

[246] Liu, S. J. et al. CRISPRi-based genome-scale identification of functional long noncoding RNA loci in human cells. *Science* **355**, eaah7111 (2016). 10.1126/science.aah7111 PMC5394926 27980086

[247] Zhu, S. et al. Genome-scale deletion screening of human long non-coding RNAs using a paired-guide RNA CRISPR-Cas9 library. *Nat. Biotechnol*. **34**, 1279–1286 (2016). 10.1038/nbt.3715 PMC5592164 27798563

[248] Esposito R (2019). Hacking the cancer genome: profiling therapeutically actionable long non-coding RNAs using CRISPR-Cas9 screening. Cancer Cell.

[249] Boettcher Michael, Tian Ruilin, Blau James A, Markegard Evan, Wagner Ryan T, Wu David, Mo Xiulei, Biton Anne, Zaitlen Noah, Fu Haian, McCormick Frank, Kampmann Martin, McManus Michael T (2018). Dual gene activation and knockout screen reveals directional dependencies in genetic networks. Nature Biotechnology.

[250] Yuan Zigao, Chen Shaopeng, Sun Qinsheng, Wang Ning, Li Dan, Miao Shuangshuang, Gao Chunmei, Chen Yuzong, Tan Chunyan, Jiang Yuyang (2017). Olaparib hydroxamic acid derivatives as dual PARP and HDAC inhibitors for cancer therapy. Bioorganic & Medicinal Chemistry.

[251] Shen, J. P. et al. Combinatorial CRISPR-Cas9 screens for de novo mapping of genetic interactions. *Nat. Methods* **14***,* 573–576 (2017). 10.1038/nmeth.4225 PMC5449203 28319113

[252] Baliou, S. et al. CRISPR therapeutic tools for complex genetic disorders and cancer (Review). *Int. J. Oncol* **53**. 443–468 (2018). 10.3892/ijo.2018.4434 PMC6017271 29901119

[253] Shalem O, Sanjana NE, Zhang F (2015). High-throughput functional genomics using CRISPR-Cas9. Nat. Rev. Genet..

[254] Heigwer F (2016). CRISPR library designer (CLD): software for multispecies design of single guide RNA libraries. Genome Biol..

[255] Ruiz S (2016). A Genome-wide CRISPR Screen Identifies CDC25A as a determinant of sensitivity to ATR inhibitors. Mol. Cell.

[256] Krall, E. B. et al. KEAP1 loss modulates sensitivity to kinase targeted therapy in lung cancer. *Elife* **6**, e18970 (2017). 10.7554/eLife.18970 PMC5305212 28145866

[257] Manguso, R. T. et al. In vivo CRISPR screening identifies Ptpn2 as a cancer immunotherapy target. *Nature* **547**, 413–418 (2017). 10.1038/nature23270 PMC5924693 28723893

[258] Flynn R (2015). CRISPR-mediated genotypic and phenotypic correction of a chronic granulomatous disease mutation in human iPS cells. Exp. Hematol..

[259] Rahman N (2014). Mainstreaming genetic testing of cancer predisposition genes. Clin. Med.

[260] Gootenberg Jonathan S., Abudayyeh Omar O., Lee Jeong Wook, Essletzbichler Patrick, Dy Aaron J., Joung Julia, Verdine Vanessa, Donghia Nina, Daringer Nichole M., Freije Catherine A., Myhrvold Cameron, Bhattacharyya Roby P., Livny Jonathan, Regev Aviv, Koonin Eugene V., Hung Deborah T., Sabeti Pardis C., Collins James J., Zhang Feng (2017). Nucleic acid detection with CRISPR-Cas13a/C2c2. Science.

[261] Chen Janice S., Ma Enbo, Harrington Lucas B., Da Costa Maria, Tian Xinran, Palefsky Joel M., Doudna Jennifer A. (2018). CRISPR-Cas12a target binding unleashes indiscriminate single-stranded DNase activity. Science.

[262] Chertow DS (2018). Next-generation diagnostics with CRISPR. Science.

[263] Yang H (2018). Break breast cancer addiction by CRISPR/Cas9 genome editing. J. Cancer.

[264] Wang Y (2016). The BRCA1-Δ11q alternative splice isoform bypasses germline mutations and promotes therapeutic resistance to PARP inhibition and cisplatin. Cancer Res..

[265] Cyranoski D (2016). CRISPR gene-editing tested in a person for the first time. Nature.

[266] Lázaro C, Ravella A, Gaona A, Volpini V, Estivill X (1994). Neurofibromatosis type 1 due to germ-line mosaicism in a clinically normal father. N. Engl. J. Med..

[267] Wilen CB (2011). Engineering HIV-resistant human CD4+ T cells with CXCR4-specific zinc-finger nucleases. PLoS Pathog..

[268] Lunzen JV (2007). Transfer of autologous gene-modified T cells in HIV-infected patients with advanced immunodeficiency and drug-resistant virus. Mol. Ther..

[269] Voit RA, McMahon MA, Sawyer SL, Porteus MH (2013). Generation of an HIV resistant T-cell line by targeted “stacking” of restriction factors. Mol. Ther..

[270] Tebas P (2014). Gene editing of CCR5 in autologous CD4 T cells of persons infected with HIV. N. Engl. J. Med..

[271] Xu Lei, Wang Jun, Liu Yulin, Xie Liangfu, Su Bin, Mou Danlei, Wang Longteng, Liu Tingting, Wang Xiaobao, Zhang Bin, Zhao Long, Hu Liangding, Ning Hongmei, Zhang Yufeng, Deng Kai, Liu Lifeng, Lu Xiaofan, Zhang Tong, Xu Jun, Li Cheng, Wu Hao, Deng Hongkui, Chen Hu (2019). CRISPR-Edited Stem Cells in a Patient with HIV and Acute Lymphocytic Leukemia. New England Journal of Medicine.

[272] Choi KD (2009). Hematopoietic and endothelial differentiation of human induced pluripotent stem cells. Stem Cells.

[273] Dolan G (2018). Haemophilia B: Where are we now and what does the future hold?. Blood Rev..

[274] Origa R (2017). Beta-thalassemia. Genet Med.

[275] Bauer DE, Orkin SH (2015). Hemoglobin switching’s surprise: the versatile transcription factor BCL11A is a master repressor of fetal hemoglobin. Curr. Opin. Genet. Dev..

[276] Moore CBT, Christie KA, Marshall J, Nesbit MA (2018). Personalised genome editing—the future for corneal dystrophies. Prog. Retin Eye Res..

[277] Wen WS, Yuan ZM, Ma SJ, Xu J, Yuan DT (2016). CRISPR-Cas9 systems: versatile cancer modelling platforms and promising therapeutic strategies. Int. J. Cancer.

[278] Fu Y (2013). High-frequency off-target mutagenesis induced by CRISPR-Cas nucleases in human cells. Nat. Biotechnol..

[279] Guilinger JP (2014). Broad specificity profiling of TALENs results in engineered nucleases with improved DNA-cleavage specificity. Nat. Methods.

[280] Pattanayak V, Ramirez CL, Joung JK, Liu DR (2011). Revealing off-target cleavage specificities of zinc-finger nucleases by in vitro selection. Nat. Methods.

[281] Vouillot L, Thélie A, Pollet N (2015). Comparison of T7E1 and surveyor mismatch cleavage assays to detect mutations triggered by engineered nucleases. G3 (Bethesda).

[282] Pattanayak V (2013). High-throughput profiling of off-target DNA cleavage reveals RNA-programmed Cas9 nuclease specificity. Nat. Biotechnol..

[283] Seeger, C. & Sohn, J. A. Complete spectrum of CRISPR/Cas9-induced mutations on HBV cccDNA. *Mol. Ther* **24**, 1258–1266 (2016). 10.1038/mt.2016.94 PMC5088770 27203444

[284] Gabriel R (2011). An unbiased genome-wide analysis of zinc-finger nuclease specificity. Nat. Biotechnol..

[285] Osborn Mark J, Webber Beau R, Knipping Friederike, Lonetree Cara-lin, Tennis Nicole, DeFeo Anthony P, McElroy Amber N, Starker Colby G, Lee Catherine, Merkel Sarah, Lund Troy C, Kelly-Spratt Karen S, Jensen Michael C, Voytas Daniel F, von Kalle Christof, Schmidt Manfred, Gabriel Richard, Hippen Keli L, Miller Jeffrey S, Scharenberg Andrew M, Tolar Jakub, Blazar Bruce R (2016). Evaluation of TCR Gene Editing Achieved by TALENs, CRISPR/Cas9, and megaTAL Nucleases. Molecular Therapy.

[286] Hsu PD (2013). DNA targeting specificity of RNA-guided Cas9 nucleases. Nat. Biotechnol..

[287] Mali P (2013). CAS9 transcriptional activators for target specificity screening and paired nickases for cooperative genome engineering. Nat. Biotechnol..

[288] Zetsche B, Volz SE, Zhang F (2015). A split-Cas9 architecture for inducible genome editing and transcription modulation. Nat. Biotechnol..

[289] Moon, S. B., Kim, D. Y., Ko, J. H., Kim, J. S. & Kim, Y. S. Improving CRISPR Genome Editing by Engineering Guide RNAs. *Trends Biotechnol* **37**, 870–882 (2019). 10.1016/j.tibtech.2019.01.009 30846198

[290] Cho SW (2014). Analysis of off-target effects of CRISPR/Cas-derived RNA-guided endonucleases and nickases. Genome Res..

[291] Xie S, Shen B, Zhang C, Huang X, Zhang Y (2014). sgRNAcas9: a software package for designing CRISPR sgRNA and evaluating potential off-target cleavage sites. PLoS ONE.

[292] Sander JD (2010). ZiFiT (Zinc Finger Targeter): an updated zinc finger engineering tool. Nucleic Acids Res..

[293] Kiani S (2014). CRISPR transcriptional repression devices and layered circuits in mammalian cells. Nat. Methods.

[294] Slaymaker I. M., Gao L., Zetsche B., Scott D. A., Yan W. X., Zhang F. (2015). Rationally engineered Cas9 nucleases with improved specificity. Science.

[295] Kleinstiver Benjamin P., Pattanayak Vikram, Prew Michelle S., Tsai Shengdar Q., Nguyen Nhu T., Zheng Zongli, Joung J. Keith (2016). High-fidelity CRISPR–Cas9 nucleases with no detectable genome-wide off-target effects. Nature.

[296] Kim S, Kim D, Cho SW, Kim J, Kim JS (2014). Highly efficient RNA-guided genome editing in human cells via delivery of purified Cas9 ribonucleoproteins. Genome Res..

[297] Suresh B, Ramakrishna S, Kim H (2017). Cell-penetrating peptide-mediated delivery of Cas9 protein and guide RNA for genome editing. Methods Mol. Biol..

[298] Dong L (2019). An anti-CRISPR protein disables type V Cas12a by acetylation. Nat. Struct. Mol. Biol..

[299] Shin J (2017). Disabling Cas9 by an anti-CRISPR DNA mimic. Sci. Adv..

[300] Shrivastav M, De Haro LP, Nickoloff JA (2008). Regulation of DNA double-strand break repair pathway choice. Cell Res..

[301] Ciccia A, Elledge SJ (2010). The DNA damage response: making it safe to play with knives. Mol. Cell.

[302] Chapman JR, Taylor MR, Boulton SJ (2012). Playing the end game: DNA double-strand break repair pathway choice. Mol. Cell.

[303] Maruyama T (2015). Increasing the efficiency of precise genome editing with CRISPR-Cas9 by inhibition of nonhomologous end joining. Nat. Biotechnol..

[304] Chu, V. T. et al. Increasing the efficiency of homology-directed repair for CRISPR-Cas9-induced precise gene editing in mammalian cells. *Nat. Biotechnol*. **33**, 543–548 (2015). 10.1038/nbt.3198 25803306

[305] Canny Marella D, Moatti Nathalie, Wan Leo C K, Fradet-Turcotte Amélie, Krasner Danielle, Mateos-Gomez Pedro A, Zimmermann Michal, Orthwein Alexandre, Juang Yu-Chi, Zhang Wei, Noordermeer Sylvie M, Seclen Eduardo, Wilson Marcus D, Vorobyov Andrew, Munro Meagan, Ernst Andreas, Ng Timothy F, Cho Tiffany, Cannon Paula M, Sidhu Sachdev S, Sicheri Frank, Durocher Daniel (2017). Inhibition of 53BP1 favors homology-dependent DNA repair and increases CRISPR–Cas9 genome-editing efficiency. Nature Biotechnology.

[306] Song J (2016). RS-1 enhances CRISPR/Cas9- and TALEN-mediated knock-in efficiency. Nat. Commun..

[307] Gao Y (2000). Interplay of p53 and DNA-repair protein XRCC4 in tumorigenesis, genomic stability and development. Nature.

[308] Moshous D (2001). Artemis, a novel DNA double-strand break repair/V(D)J recombination protein, is mutated in human severe combined immune deficiency. Cell.

[309] Shah SZ (2019). Advances In research on genome editing Crispr-Cas9 technology. J. Ayub Med Coll. Abbottabad.

[310] Lin S, Staahl BT, Alla RK, Doudna JA (2014). Enhanced homology-directed human genome engineering by controlled timing of CRISPR/Cas9 delivery. Elife.

[311] Kim Kyoungmi, Ryu Seuk-Min, Kim Sang-Tae, Baek Gayoung, Kim Daesik, Lim Kayeong, Chung Eugene, Kim Sunghyun, Kim Jin-Soo (2017). Highly efficient RNA-guided base editing in mouse embryos. Nature Biotechnology.

[312] Zafra Maria Paz, Schatoff Emma M, Katti Alyna, Foronda Miguel, Breinig Marco, Schweitzer Anabel Y, Simon Amber, Han Teng, Goswami Sukanya, Montgomery Emma, Thibado Jordana, Kastenhuber Edward R, Sánchez-Rivera Francisco J, Shi Junwei, Vakoc Christopher R, Lowe Scott W, Tschaharganeh Darjus F, Dow Lukas E (2018). Optimized base editors enable efficient editing in cells, organoids and mice. Nature Biotechnology.

[313] Grünewald Julian, Zhou Ronghao, Iyer Sowmya, Lareau Caleb A., Garcia Sara P., Aryee Martin J., Joung J. Keith (2019). CRISPR DNA base editors with reduced RNA off-target and self-editing activities. Nature Biotechnology.

[314] Zong Yuan, Song Qianna, Li Chao, Jin Shuai, Zhang Dingbo, Wang Yanpeng, Qiu Jin-Long, Gao Caixia (2018). Efficient C-to-T base editing in plants using a fusion of nCas9 and human APOBEC3A. Nature Biotechnology.

[315] Hur Junho K, Kim Kyoungmi, Been Kyung Wook, Baek Gayoung, Ye Sunghyeok, Hur Junseok W, Ryu Seuk-Min, Lee Youn Su, Kim Jin-Soo (2016). Targeted mutagenesis in mice by electroporation of Cpf1 ribonucleoproteins. Nature Biotechnology.

[316] Gori Jennifer L., Hsu Patrick D., Maeder Morgan L., Shen Shen, Welstead G. Grant, Bumcrot David (2015). Delivery and Specificity of CRISPR/Cas9 Genome Editing Technologies for Human Gene Therapy. Human Gene Therapy.

[317] Zuris JA (2015). Cationic lipid-mediated delivery of proteins enables efficient protein-based genome editing in vitro and in vivo. Nat. Biotechnol..

[318] Mout, R., Ray, M., Lee, Y. W., Scaletti, F. & Rotello, V. M. In vivo delivery of CRISPR/Cas9 for therapeutic gene editing: progress and challenges. *Bioconjug. Chem*. **28**, 880–884 (2017). 10.1021/acs.bioconjchem.7b00057 PMC5846329 28263568

[319] Yin H, Kauffman KJ, Anderson DG (2017). Delivery technologies for genome editing. Nat. Rev. Drug Disco..

[320] Kotterman MA, Schaffer DV (2014). Engineering adeno-associated viruses for clinical gene therapy. Nat. Rev. Genet..

[321] Maggio I (2014). Adenoviral vector delivery of RNA-guided CRISPR/Cas9 nuclease complexes induces targeted mutagenesis in a diverse array of human cells. Sci. Rep..

[322] Feng M (1997). Stable in vivo gene transduction via a novel adenoviral/retroviral chimeric vector. Nat. Biotechnol..

[323] Koike-Yusa H, Li Y, Tan EP, Velasco-Herrera Mdel C, Yusa K (2014). Genome-wide recessive genetic screening in mammalian cells with a lentiviral CRISPR-guide RNA library. Nat. Biotechnol..

[324] Paulk NK (2010). Adeno-associated virus gene repair corrects a mouse model of hereditary tyrosinemia in vivo. Hepatology.

[325] Charlesworth, C. T. et al. Identification of pre-existing adaptive immunity to Cas9 proteins in humans. *bioRxiv*10.1101/243345 (2018).

[326] Ihry, R. J. et al. p53 inhibits CRISPR-Cas9 engineering in human pluripotent stem cells. *Nat. Med*. **24**, 939–946 (2018). 10.1038/s41591-018-0050-6 29892062

[327] Liang C (2017). Tumor cell-targeted delivery of CRISPR/Cas9 by aptamer-functionalized lipopolymer for therapeutic genome editing of VEGFA in osteosarcoma. Biomaterials.

[328] Luo Ying-Li, Xu Cong-Fei, Li Hong-Jun, Cao Zhi-Ting, Liu Jing, Wang Ji-Long, Du Xiao-Jiao, Yang Xian-Zhu, Gu Zhen, Wang Jun (2018). Macrophage-Specific in Vivo Gene Editing Using Cationic Lipid-Assisted Polymeric Nanoparticles. ACS Nano.

[329] Finn JD (2018). A single administration of CRISPR/Cas9 lipid nanoparticles achieves robust and persistent in vivo genome editing. Cell Rep..

[330] Wang HX (2018). Nonviral gene editing via CRISPR/Cas9 delivery by membrane-disruptive and endosomolytic helical polypeptide. Proc. Natl Acad. Sci. USA.

[331] Ramakrishna S (2014). Gene disruption by cell-penetrating peptide-mediated delivery of Cas9 protein and guide RNA. Genome Res..

[332] Ma Y (2016). Increasing the efficiency of CRISPR/Cas9-mediated precise genome editing in rats by inhibiting NHEJ and using Cas9 protein. RNA Biol..

[333] Guenther CM (2014). Synthetic virology: engineering viruses for gene delivery. Wiley Interdiscip. Rev. Nanomed. Nanobiotechnol..

[334] Cronin J, Zhang XY, Reiser J (2005). Altering the tropism of lentiviral vectors through pseudotyping. Curr. Gene Ther..

[335] Ho ML (2014). Efficiency of protease-activatable virus nanonodes tuned through incorporation of wild-type capsid subunits. Cell. Mol. Bioeng..

[336] Asuri P (2012). Directed evolution of adeno-associated virus for enhanced gene delivery and gene targeting in human pluripotent stem cells. Mol. Ther..

[337] Hofherr SE, Mok H, Gushiken FC, Lopez JA, Barry MA (2007). Polyethylene glycol modification of adenovirus reduces platelet activation, endothelial cell activation, and thrombocytopenia. Hum. Gene Ther..

[338] Lee GK, Maheshri N, Kaspar B, Schaffer DV (2005). PEG conjugation moderately protects adeno-associated viral vectors against antibody neutralization. Biotechnol. Bioeng..

[339] Kay MA, He CY, Chen ZY (2010). A robust system for production of minicircle DNA vectors. Nat. Biotechnol..

[340] Koo Taeyoung, Yoon A-Rum, Cho Hee-Yeon, Bae Sangsu, Yun Chae-Ok, Kim Jin-Soo (2017). Selective disruption of an oncogenic mutant allele by CRISPR/Cas9 induces efficient tumor regression. Nucleic Acids Research.

[341] Lee K (2017). Nanoparticle delivery of Cas9 ribonucleoprotein and donor DNA in vivo induces homology-directed DNA repair. Nat. Biomed. Eng..

